# Coordination of Glucosinolate Biosynthesis and Turnover Under Different Nutrient Conditions

**DOI:** 10.3389/fpls.2019.01560

**Published:** 2019-12-06

**Authors:** Verena Jeschke, Konrad Weber, Selina Sterup Moore, Meike Burow

**Affiliations:** DynaMo Center, Department of Plant and Environmental Sciences, University of Copenhagen, Frederiksberg, Denmark

**Keywords:** glucosinolate metabolism, nutrient conditions, seedling development, nitrogen limitation, sulphur limitation, metabolic regulation

## Abstract

Dynamically changing environmental conditions promote a complex regulation of plant metabolism and balanced resource investments to development and defense. Plants of the Brassicales order constitutively allocate carbon, nitrogen, and sulfur to synthesize glucosinolates as their primary defense metabolites. Previous findings support a model in which steady-state levels of glucosinolates in intact tissues are determined by biosynthesis and turnover through a yet uncharacterized turnover pathway. To investigate glucosinolate turnover in the absence of tissue damage, we quantified exogenously applied allyl glucosinolate and endogenous glucosinolates under different nutrient conditions. Our data shows that, in seedlings of *Arabidopsis thaliana* accession Columbia-0, glucosinolate biosynthesis and turnover are coordinated according to nutrient availability. Whereas exogenous carbon sources had general quantitative effects on glucosinolate accumulation, sulfur or nitrogen limitation resulted in distinct changes in glucosinolate profiles, indicating that these macronutrients provide different regulatory inputs. Raphanusamic acid, a breakdown product that can potentially be formed from all glucosinolate structures appears not to reflect *in planta* turnover rates, but instead correlates with increased accumulation of endogenous glucosinolates. Thus, raphanusamic acid could represent a metabolic checkpoint that allows glucosinolate-producing plants to measure the flux through the biosynthetic and/or turnover pathways and thereby to dynamically adjust glucosinolate accumulation in response to internal and external signals.

## Introduction

Plants rely on a multitude of constitutive and inducible specialized metabolites that mediate interactions with the environment. These range from deterring pathogen and herbivores, over attracting beneficial organisms and enemies, to providing protection from, for example, UV radiation or drought. The chemodiversity of specialized metabolites and their regulation in terms of quantity and composition across tissues and developmental stages is beyond comparison (Moore et al., 2014). *Arabidopsis thaliana*, a small herbaceous plant of the Brassicaceae family, is a widely used and well-studied model organism for plant specialized metabolism, particularly the metabolism of glucosinolates (D’Auria and Gershenzon, 2005; Jensen et al., 2014; Burow and Halkier, 2017). Glucosinolates are chemically diverse amino acid-derived, sulfur- and nitrogen-containing thioglucosides almost solely found in plants of the order Brassicales with more than 130 identified structures to date (Blažević et al., 2019). The activation of glucosinolates has been intensively studied, both for their defensive roles against pathogens and herbivores and for their health beneficial roles for humans (Burow et al., 2010; Pastorczyk and Bednarek, 2016; Traka, 2016; Wittstock et al., 2016a). In recent years, more and more studies uncovering additional roles of glucosinolates and glucosinolate-derived metabolites in feedback regulation of plant metabolism, growth and defense emerged (Zhao et al., 2008; Kerwin et al., 2011; Burow et al., 2015; Jensen et al., 2015; Katz et al., 2015; Francisco et al., 2016a; Malinovsky et al., 2017; Urbancsok et al., 2017; Urbancsok et al., 2018). These studies have connected primary and specialized metabolism *via* glucosinolate-mediated signaling networks, and provided first insights into the regulatory interplay between glucosinolate metabolism and plant development.

As precursors of defensive metabolites, glucosinolates are constitutively biosynthesized in almost all tissues of *A. thaliana* throughout all developmental stages of the plant. Sufficient amounts of glucosinolates must be present in a given tissue for the glucosinolate-myrosinase system to function as defense. In the flower stalk, S-cells store glucosinolates accounting for 40% of the total sulfur in that tissue and these have also been identified in petioles (Koroleva et al., 2000; Koroleva et al., 2010). A considerable proportion of glucosinolates thus seems to be stored in these idioblasts. Nevertheless, glucosinolate quantity and composition vary dynamically depending on the age of the plant, the tissue studied and the environmental conditions (Petersen et al., 2002; Brown et al., 2003; Kliebenstein et al., 2005; Mewis et al., 2005; Gigolashvili et al., 2009; Textor and Gershenzon, 2009; Mewis et al., 2012; Züst et al., 2012; Huseby et al., 2013; Augustine and Bisht, 2016; Burow, 2016). Furthermore, a growing number of studies have identified additional functions of glucosinolates; often of distinct of glucosinolate structures, that are not directly linked to plant defense, but instead to hormone signaling, stomatal aperture, the circadian clock, root growth, biomass, and the onset of flowering (Zhao et al., 2008; Kerwin et al., 2011; Burow et al., 2015; Jensen et al., 2015; Katz et al., 2015; Francisco et al., 2016a; Francisco et al., 2016b; Malinovsky et al., 2017; Urbancsok et al., 2017; Urbancsok et al., 2018). These observations illustrate how intricately glucosinolate metabolism connects to a multitude of metabolic and developmental processes; and they may explain the dynamic nature of glucosinolate profiles, also in the absence of a biotic attacker.

Glucosinolate content reaches levels of up to 3% of the dry weight (Brown et al., 2003; Falk et al., 2007) making it a potentially costly investment for the plant (Bekaert et al., 2012; Züst and Agrawal, 2017). Because glucosinolates contain sulfur and nitrogen, their metabolism is tightly linked with sulfur and nitrogen assimilation and metabolism (Kopriva and Gigolashvili, 2016; Marino et al., 2016). Changes in abundance and composition depending on sulfur and nitrogen availability has been intensely studied in *A. thaliana* and glucosinolate-producing vegetables. Generally, sulfur availability and assimilation is positively correlated with aliphatic glucosinolate levels, while nitrogen availability is positively correlated with indolic glucosinolate levels [for example: (Booth et al., 1991; Withers and O’Donnell, 1994; Koprivova et al., 2000; Krumbein et al., 2001; Kopsell et al., 2007; Omirou et al., 2009; Frerigmann and Gigolashvili, 2014)]. However, increasing nitrogen concentrations have also been shown to have negative effects on total and aliphatic glucosinolate levels depending on the proportion of other macronutrients present such as sulfur, potassium and phosphorous (Li et al., 2007; Schonhof et al., 2007; Chun et al., 2017). Further, the type of nitrogen source (nitrate versus ammonium) can affect glucosinolate levels (Marino et al., 2016). Besides aforementioned inorganic macronutrients, different sugars have been shown to have a positive effect on glucosinolate levels in broccoli sprouts (Guo et al., 2011). Up to 30% of the total sulfur pool are bound in glucosinolates, which were therefore discussed as potential sulfur storage that can be remobilized under conditions of sulfur deficiency or starvation (Falk et al., 2007). The general ability of *A. thaliana* to metabolize glucosinolates has been directly demonstrated. When *p*-hydroxybenzyl glucosinolate (sinalbin) was provided as the only sulfur source, seedlings accumulated higher levels of sulfur-containing metabolites, including glucosinolates, than seedlings on glucosinolate-free, sulfur-deficient medium (Zhang et al., 2011). However, endogenous glucosinolates were shown not to be a sulfur source in Brassica seedlings when other sources of organic sulfur were present (Aghajanzadeh et al., 2014). This suggests that seedlings are capable of utilizing glucosinolates as a nutrient under conditions of deficiency, but glucosinolates are not a preferred source for sulfur remobilization. Unlike cyanogenic glycosides (Pičmanová et al., 2015), glucosinolates have not been investigated for a potential function as storage form of nitrogen.

**Figure 1:**
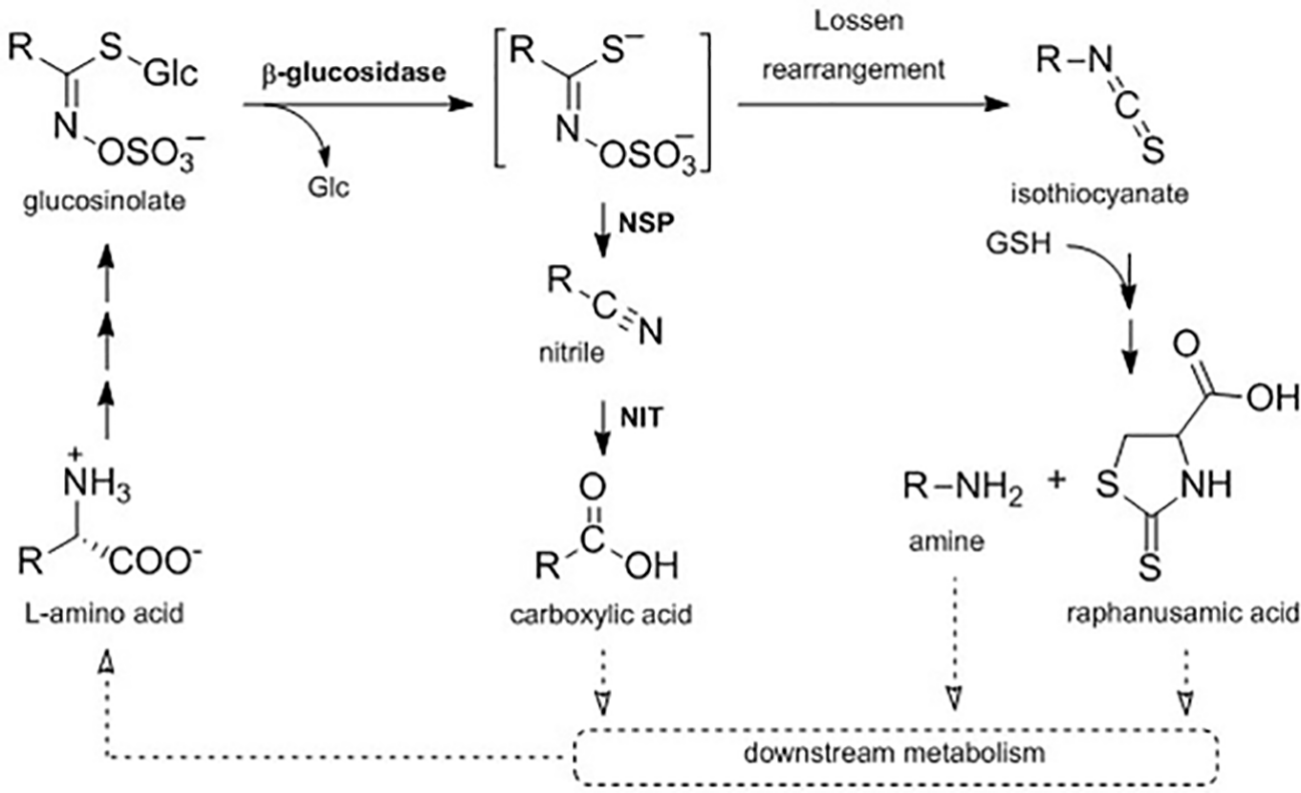
A proposed metabolic pathway connecting primary and specialized metabolism in intact *A. thaliana* Col-0 seedlings. *In planta* turnover of glucosinolates may proceed *via* hydrolytic cleavage of the glucose (Glc) moiety by a ß-glucosidase to form an aglucone, which is either metabolized to the corresponding nitrile in dependency of nitrile-specifier proteins (NSPs) or rearranged to the isothiocyanate. Nitriles can be further metabolized by nitrilases (NIT) to carboxylic acids, while isothiocyanates can be conjugated to glutathione (GSH) and metabolized to the corresponding amine and raphanusamic acid. Further downstream metabolization of those metabolites is not yet elucidated (dotted arrows).

Studies on glucosinolate breakdown have traditionally focused on the enzymatic activation of glucosinolates to bioactive compounds initiated by myrosinases upon tissue damage, i.e. caused by feeding insects (Matile, 1980; Kissen et al., 2009; Wittstock and Burow, 2010). Turnover in intact tissue (i.e. their *in planta* metabolization in absence of tissue-damage induced breakdown) is less well understood and the pathways and their regulation are yet to be uncovered (**Figure 1**). Earlier studies that investigated the dynamics of glucosinolates in *A. thaliana* during plant development or in adult rosettes at different times of day suggested turnover processes in concurrence with *de novo* biosynthesis (Petersen et al., 2002; Brown et al., 2003; Huseby et al., 2013). For example, relative and absolute amounts of glucosinolates change considerably during the transitions from seeds to seedlings and from seedlings to adult rosette (Brown et al., 2003). Furthermore, in *A. thaliana* Col-0, some methionine-derived glucosinolates (embryo-synthesized glucosinolates, **Supplementary Figure 1**) are only present in seeds and seedlings but not in vegetative rosette tissue of young plants indicative of *in planta* turnover of glucosinolates in seedlings (Petersen et al., 2002; Brown et al., 2003). Indeed, labeled [^14^C]*p*-hydroxybenzyl glucosinolate fed to rosette leaves of flowering plants accumulated in the seeds and subsequently showed a decline to 70% within the first weeks of plant development in the next generation (Petersen et al., 2002).

It is currently unknown which enzymes and pathways are involved in *in planta* turnover of glucosinolates. Hydrolysis of the thioglucosidic bond by a β-glucosidase has been proposed as the first step (**Figure 1**). Experimental evidence suggests that the classical myrosinases TGG1 and TGG2 are not involved in glucosinolate turnover in the absence of tissue damage (Barth and Jander, 2006), but in total, 40 family 1 β-glucosidases are encoded in the *A. thaliana* Col-0 genome (Xu et al., 2004). For three of these enzymes besides classical myrosinases, it has been shown that they can hydrolyze glucosinolates under certain conditions and in certain tissues (Bednarek et al., 2009; Nakano et al., 2017; Nakazaki et al., 2019). In *A. thaliana* Col-0, damage-induced glucosinolate activation by myrosinases results in the formation of aglucones that can either arrange to isothiocyanates or be converted into simple nitriles (**Figure 1**) (Wittstock and Burow, 2007; Wittstock and Burow, 2010; Wittstock et al., 2016a). Nitrile-formation is dependent on action of nitrile-specifier proteins (NSPs), which are expressed in seeds, seedlings, leaves, and roots with an organ-specific regulation in *A. thaliana* Col-0 (Burow et al., 2009; Wittstock et al., 2016b). Glucosinolate-derived nitriles were suggested to be further converted to the corresponding carboxylic acids by nitrilases (NITs), yet these conversions were not considered to contribute to growth regulation *via* auxin signaling (Piotrowski, 2008; Janowitz et al., 2009). More recently, *in silico* modelling did, however, not hint to a major impact of nitrilase activity on auxin signaling (Vik et al., 2018). Spontaneous rearrangement of the aglucones yields isothiocyanates, which are highly reactive electrophiles (Brown and Hampton, 2011). Upon activation of 4-methoxyindol-3-ylmethyl glucosinolate (4MOI3M) by the atypical myrosinase PEN2, the resulting isothiocyanate is conjugated with glutathione and undergoes further metabolization to the corresponding amine bearing the indolic side-chain, a reaction that also yields the sulfur-containing raphanusamic acid (**Figure 1**) (Bednarek et al., 2009; Piślewska-Bednarek et al., 2018). Raphanusamic acid has been reported to have inhibitory properties on plant growth (Inamori et al., 1992) and to be involved in plant immunity (Bednarek, 2012), but the *in planta* mode of action remained elusive.

Here, to investigate the dynamics of glucosinolate biosynthesis and turnover in seedlings of *A. thaliana* Col-0 in the absence of tissue damage, we quantitatively analyzed the profiles of endogenous glucosinolates and the dynamics of the exogenous allyl glucosinolate under different nutrient conditions, specifically sulfur and nitrogen limitation as well as availability of exogenous sugars. Our data shows that glucosinolate biosynthesis and its downstream *in planta* turnover are coordinated according to nutrient availability. Under our experimental conditions, raphanusamic acid accumulation correlated with glucosinolate accumulation, suggesting that this turnover pathway intermediate can provide the plant with information on total glucosinolate levels.

## Materials and Methods

### Plant Treatments

*A. thaliana* seedling Col-0 (NASC, N1093) were sterilized with chlorine gas (generated by mixing 100 ml 14% sodium hypochlorite and 3 ml 37% HCL) for 3h. Growth medium was prepared as described in a published protocol (dx.doi.org/10.17504/protocols.io.5q6g5ze). Total nitrogen concentrations were 3 mM (+N) and 0.3 mM (−N) and total sulfur concentrations 1.68 mM (+S) and 0.015 mM (−S). ½ MS medium (**Figure 3B**) was purchased from Duchefa Biochemie (product no. M0222) and had a total sulfur concentration of 0.82 mM and total nitrogen concentration of 30 mM. Sugars were added to the growth medium solution prior to autoclaving. The concentrations of sugars used correspond to 1% (w/v, 29.2 mM) and 2% (w/v, 58.4 mM) for the monosaccharides glucose and fructose and the sugar alcohols mannitol and sorbitol, and 1.9% (w/v, 29.2 mM) and 3.8% (w/v, 58.4 mM) for the disaccharide sucrose. To study allyl glucosinolate accumulation and turnover, 100 mM allyl glucosinolate (Sinigrin, Sigma-Aldrich, No. S1647) stock solution in water was added to 50 ml of the autoclaved hand warm medium to the final concentrations indicated in the figures and carefully mixed by inversion. For all media treatments, 50 ml medium was poured into a square plate (120 ×120 × 16 mm, Frisenette, Denmark) and the medium was allowed to cool. After plating of the seeds, plates were placed for four days in the dark at 4°C for stratification and subsequently moved to a growth chamber (16 h light/8 h dark, 22/21°C, average light intensity 160 µE), in a vertical orientation. Germination was scored daily as the emergence of the radicle. Only seedlings that germinated one day after stratification were considered for further analysis. To investigate turnover of glucosinolates under different nutrient conditions, seedlings were carefully transferred after phase 1 (accumulation of exogenously applied allyl glucosinolate) using soft forceps onto plates containing different media compositions but no allyl glucosinolate (phase 2) (**Supplementary Figure 3**). Seedling weight was recorded for a single seedling using a fine-balance prior to metabolite sampling if indicated.

### Metabolite Analyses

For glucosinolate and raphanusamic acid analysis, seedlings were harvested into 300 µl 85% (v/v) methanol (HPLC grade) containing 10 µM *p*-hydroxybenzyl glucosinolate (pOHb; PhytoLab, cat. No. 89793) as internal standard and subsequently homogenized in a bead mill (3 mm bearing balls, 2 × 30 s at 30 Hz). After centrifugation (5 min, 4,700×*g*, 4°C), 20 µl of the supernatant was diluted with 180 µL MilliQ-grade water (1:10) and filtered (Durapore^®^ 0.22 µm PVDF filters, Merck Millipore, Tullagreen, Ireland) for the analysis of raphanusamic acid. For glucosinolate analysis, samples were prepared as desulfo-glucosinolates as previously described (alternate protocol 2, Crocoll et al., 2016) and analysed with LC-MS/MS. LC-MS/MS analysis was carried out on an Advance UHPLC system (Bruker, Bremen, Germany) equipped with a Kinetex^®^ XB-C18 column (100 × 2.1 mm, 1.7 µm, 100 Å, Phenomenex, USA) coupled to an EVOQ Elite TripleQuad mass spectrometer (Bruker, Bremen, Germany) equipped with an electrospray ionization source (ESI). The injection volume was 1 µL. Separation was achieved with a gradient of water/0.05% (v/v) formic acid (solvent A)–acetonitrile (solvent B) at a flow rate of 0.4 ml/min at 40°C (formic acid, Sigma-Aldrich, cat. no. F0507, reagent grade; acetonitrile, HPLC grade). The elution profile was: 0–0.5 min 2% B; 0.5–1.2 min 2-30% B; 1.2–2.0 min 30–100% B; 2.0–2.5 min 100% B; 2.5–2.6 min 100–2% B; 2.6–4.0 min 2% B. For raphanusamic acid the instrument parameters were optimized by infusion with the pure standard (Sigma-Aldrich, No. 273449). The mass spectrometer parameters were as follows: ionspray voltage was maintained at 3,500 V, cone temperature was set to 300°C and heated probe temperature to 400°C, cone gas flow was set to 20 psi, probe gas flow to 40 psi, nebulizer gas 60 psi, and collision gas to 1.5 mTorr. Nitrogen was used as cone and nebulizer gas, and argon as collision gas. Multiple reaction monitoring (MRM) was used to monitor analyte parent ion > product ion transitions [collision energy]: (pos) 164 > 118 [15 V] (quantifier) and 164 > 59 [15 V] (qualifier). Both Q1 and Q3 quadrupoles were maintained at unit resolution. Glucosinolates were quantified relative pOHb using experimentally determined response factors with commercially available standards in a representative plant matrix. Raphanusamic acid was quantified based on external calibration using a dilution series of an authentic standard.

### Statistical Analysis

Statistical analyses (parametric and non-parametric standard tests) were performed using the software R (version 3.3.2, R Core Team, 2017) after checking the suitability of the model for scedasticity and distribution of the data. Models used for analysis are indicated in the figure and supplementary table legends. Figures were generated using the package "ggplot2." MFA (multiple factor analysis) was performed and visualized using the packages "FactoMineR" and "factoextra."

## Results

### Exogenous Carbon Sources Affect Total Amounts of Glucosinolates During Seedling Development

Glucosinolate profiles differ markedly between seeds and seedlings of A. thaliana (Haughn et al., 1991; Brown et al., 2003). In the accession Col-0, glucosinolates with hydroxyalkyl and benzoyloxyalkyl side chains are synthesized in the developing seeds and still present in seedlings but not at later developmental stages (**Supplementary Figure 2**) (Kliebenstein et al., 2001; Brown et al., 2003; Kliebenstein et al., 2007). We refer here to these structures as embryo-synthesized glucosinolates. During the transition from seed to seedling, the total glucosinolate content transiently increases, but then drops again to seed level within two to six days after planting. Interestingly, the content of embryo-synthesized glucosinolates is stable during germination (Brown et al., 2003), suggesting that they are not yet catabolized at this early stage of seed-to-seedling transition. To study glucosinolate turnover kinetics, we therefore focused on seedling development after germination.

**Figure 2:**
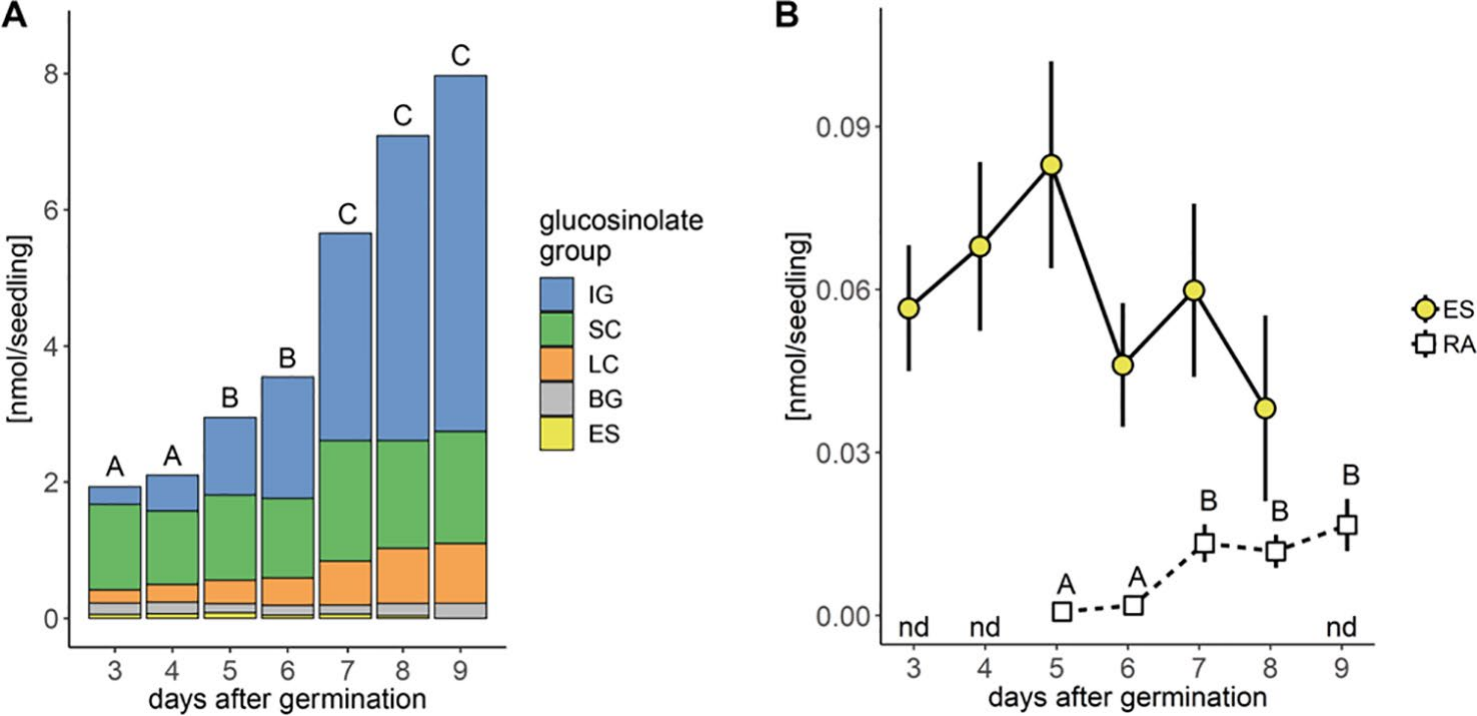
Glucosinolate accumulation during *A. thaliana* Col-0 seedling development. **(A)** Plotted are means (N = 6–18, one experimental round) of glucosinolate classes: IG, indolic glucosinolates; SC, short-chain aliphatic glucosinolates; LC, long-chain aliphatic glucosinolates; BG, benzenic glucosinolates; ES, embryo-synthesized glucosinolates. Letters denote significant differences at the level on total glucosinolate levels (P < 0.05). **(B)** Levels of embryo-synthesized glucosinolates (ES; P = 0.62) and raphanusamic acid (RA; P < 0.001). Embryo-synthesized glucosinolates were not detected (nd) in nine-day-old seedlings and raphanusamic acid was not detected in three- and four-day-old seedlings. Depicted are means ± SE, N = 6–18. Statistical testing was performed with Kruskal–Wallis rank sum test followed by pairwise comparisons using Wilcoxon rank sum test (P-value adjustment method: Benjamini & Hochberg). Details on the statistical analysis including means and standard deviations are provided in **Supplementary Table 1** for glucosinolate groups and **Supplementary Table 2** for individual glucosinolates.

**Figure 3:**
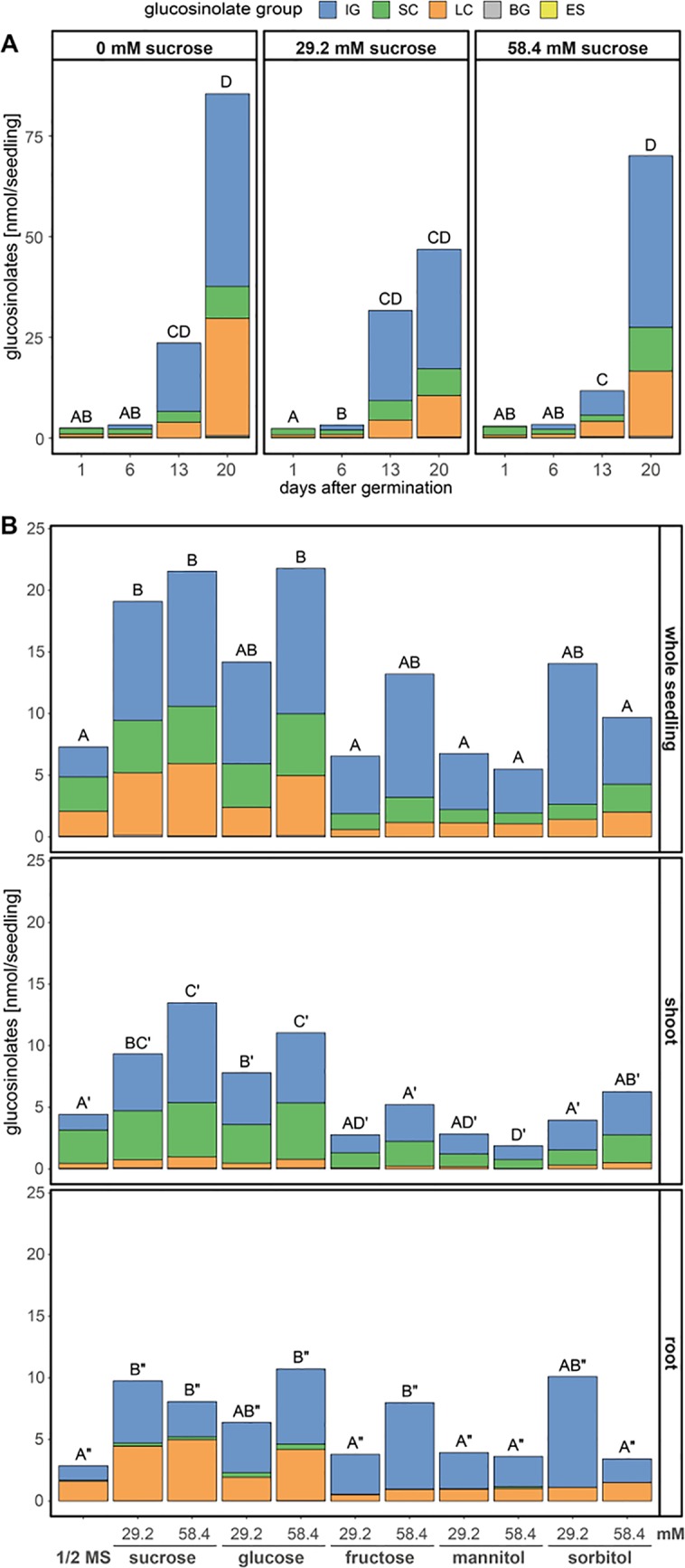
Glucosinolate accumulation in *A. thaliana* Col-0 seedlings depending on the presence and concentration of different sugars in the growth media. Plotted are the means of glucosinolate classes: IG, indolic glucosinolates; SC, short-chain aliphatic glucosinolates; LC, long-chain aliphatic glucosinolates; BG, benzenic glucosinolates; ES, embryo-synthesized glucosinolates. Letters denote significant differences at the 0.05 level on total glucosinolate levels. **(A)** Seedlings were grown on medium (1.68 mM S, 3 mM N) with three different sucrose concentrations and harvested 1–20 days after germination, (N = 6–8, one experimental round). Different letters indicate statistical differences across all treatments and days. Statistical details including means and standard deviations are in **Supplementary Table 3** for glucosinolate groups and **Supplementary Table 4** for individual glucosinolates. **(B)** Seedlings grew for twelve days after germination on ½ MS medium (0.82 mM S, 30 mM N) supplemented with different sugars, before shoot and root tissue were separately harvested per individual seedling. Letters denote significant differences of total glucosinolates levels within a tissue determined by pairwise comparison using Wilcoxon rank sum test (P-value adjustment method: Benjamini & Hochberg) (N = 12, one experimental round). Details on the statistical analysis including means and standard deviations are provided in **Supplementary Table 5** for glucosinolate groups and **Supplementary Table 6** for individual glucosinolates. Growth phenotypes are depicted in **Supplementary Figure 4**. ½ MS, half strength Murashige & Skoog medium.

We analyzed glucosinolates in individual seedlings, starting three days after germination. Overall glucosinolate content continuously increased by 4-fold within six days (P < 0.001, **Figure 2A**) and more than 30-fold within 20 days after germination (P < 0.001, **Figure 3A**). This increase affects all three main glucosinolate classes of short- and long-chain aliphatic and indolic glucosinolates but is initially mainly driven by indolic glucosinolates (most dominantly NMOI3M; **Supplementary Tables 1** and **2**) and then later also by long-chain aliphatic glucosinolates (**Supplementary Table 3**). The group of embryo-synthesized glucosinolates (**Supplementary Figure 1**), herein 3bzo and 4bzo glucosinolates, slightly decrease with seedling development (P = 0.062) until they are not detected at nine days after germination (**Figure 2B**). Interestingly, we detected relatively high levels of embryo-synthesized glucosinolates decreasing at later developmental stages in a different developmental setup (see below).

Noticeable increases of glucosinolate content between five and seven days after germination correlated with the development of the first lateral roots and the expansion of the first true leaves, respectively. Concurrent with these physiological changes in seedling development and the pattern of glucosinolate accumulation, levels of raphanusamic acid show increases at five and seven days after germination (P < 0.001, **Figure 2B**).

The addition of exogenous carbon sources to the growth medium, as frequently done in studies on *A. thaliana* seedlings, had several effects on the dynamics of glucosinolate accumulation (**Figure 3**) and seedling development (**Supplementary Figure 4**). Exogenously supplied sucrose affected the rate and timing of the accumulation of all individual glucosinolates in whole seedlings (**Figures 3A, B**, top panel, **Supplementary Table 3**). Most dominantly affected by 29.2 mM sucrose treatment were the dynamics of long-chain aliphatic and indolic glucosinolate accumulation while the changes in short-chain aliphatic glucosinolates over time seemed unaffected (**Supplementary Table 3**). Compared to sucrose treatment, the effects of glucose treatment on glucosinolate levels was similar, while that of fructose was weaker (**Figure 3B**). Twelve-day-old seedlings showed a clear distribution of short-chain aliphatic glucosinolates predominantly in shoots and long-chain aliphatic glucosinolates mostly in roots. This tissue-specific distribution remained unaffected by all sugar treatments, albeit the absolute levels and ratios between all glucosinolate groups changed depending on the sugar applied (**Figure 3B** and **Supplementary Table 5**). The tested sugars additionally had considerable growth and developmental effects (**Supplementary Figure 4**). While seedlings grown in the presence of sucrose or glucose show an increase in lateral root formation and shoot size, fructose and mannitol caused an overall stunted growth phenotype. This suggests that the increase in glucosinolates in sucrose and glucose treated seedlings relates to the increased seedling size, while fructose treated seedlings accumulate considerably lower glucosinolate contents, possibly due to their smaller size (**Figure 3B** and **Supplementary Figure 4**).

### Sulfur and Nitrogen Limitation Differentially Affects Total and Relative Glucosinolate Levels

**Figure 4:**
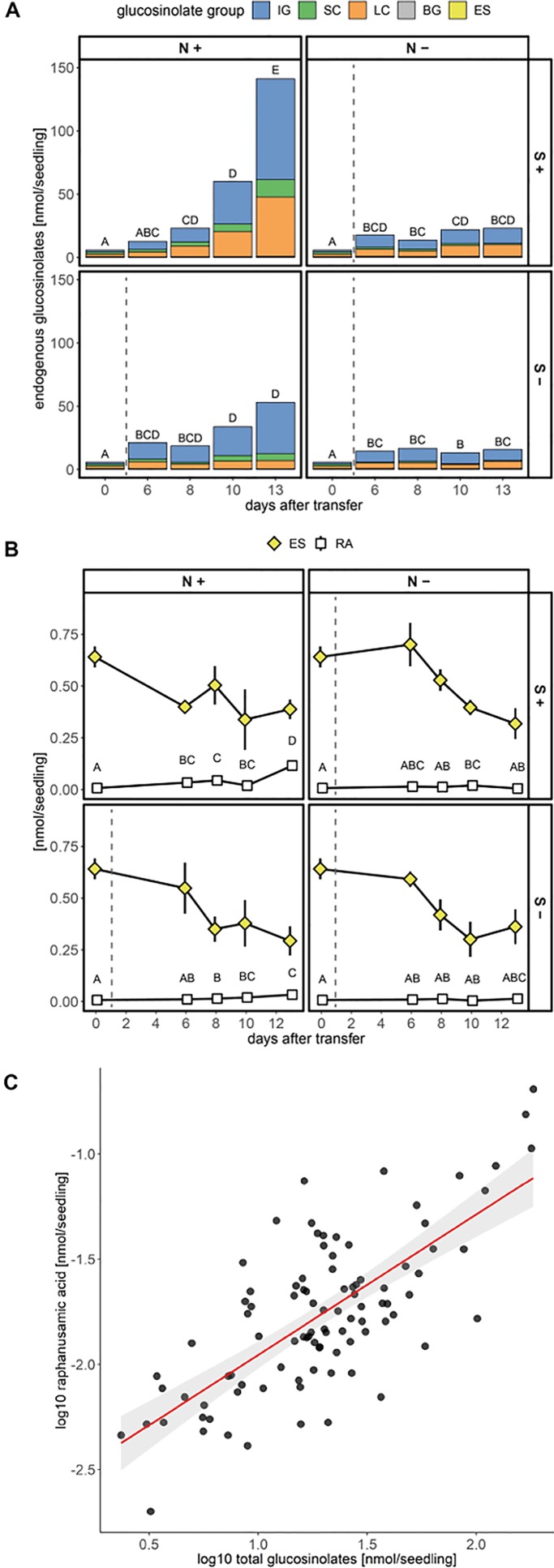
Accumulation of glucosinolates and raphanusamic acid in *A. thaliana* Col-0 seedlings under different sulfur (S) and nitrogen (N) regimes. Seeds were germinated on control medium containing sufficient amounts of sulfur (+S) and nitrogen (+N) and seedlings were grown for six days (phase 1) before they were transferred to media with varying sulfur and nitrogen nutrition (phase 2, **Supplementary Figure 3**). Day 0 describes the day of transfer and reflects the metabolite levels of six-day-old seedlings grown on +S/+N medium. The dashed grey line indicates that seedlings had a change to a limiting medium condition. **(A)** Total glucosinolate levels and **(B)** levels of embryo-synthesized glucosinolates (ES) and accumulation of raphanusamic acid (RA) under sufficient and limiting sulfur and nitrogen nutrition. Plotted are **(A)** means of the glucosinolate classes: IG, indolic glucosinolates; SC, short-chain aliphatic glucosinolates; LC, long-chain aliphatic glucosinolates; BG, benzenic glucosinolates; ES, embryo-synthesized glucosinolates, and **(B)** means ± SE (N = 5–16, two independent experiments pooled). Letters denote significant difference at the 0.05 level determined by pairwise comparison using Wilcoxon rank sum test (P-value adjustment method: Benjamini & Hochberg). Details on the statistical analysis including means and standard deviations are provided in **Supplementary Table 7** for glucosinolate groups and **Supplementary Table 8** for individual glucosinolates. Seedling fresh weight in nitrogen and/or sulfur sufficient and limiting conditions is plotted in **Supplementary Figure 5**. **(C)** Correlation between total levels of glucosinolates and raphanusamic acid for seedlings grown on sulfur and nitrogen sufficient medium harvested daily three to nine days after germination. Slope = 0.667 ± 0.066, *P* < 0.001 (F = 103.30), adj. R2 = 0.521 of the linear model lm(logRA∼logGLS). Correlations of raphanusamic acid to levels of indolic, short-chain aliphatic and long-chain aliphatic glucosinolates for seedlings grown on glucosinolate-free medium are depicted in **Supplementary Figures 5A–C**, correlations of raphanusamic acid to total glucosinolate levels and accumulated allyl glucosinolate for seedlings grown on medium supplemented with 50 μM allyl glucosinolate are depicted in **Supplementary Figures 5D–E**.

Next, we tested the effect of sulfur and nitrogen limitation, alone and in combination, on glucosinolate accumulation and growth of *A. thaliana* Col-0 seedlings for several days. Before the seedlings were exposed to different nutrient regimes, seeds were germinated and grown for six days on control medium containing sufficient amounts of sulfur and nitrogen. Seedlings were then transferred to different media limited in sulfur and/or nitrogen (**Supplementary Figure 3**) and their development and glucosinolate profile was followed for several days. Nitrogen limitation led to reduced seedling weight (**Supplementary Figure 5**) and significantly affected accumulation of all *de novo* synthesized glucosinolate classes (indolic, short- and long-chain aliphatic glucosinolates) within ten to 13 days after transfer (16 to 19 day-old seedlings) and caused stagnation in glucosinolate accumulation (**Figure 4A** and **Supplementary Table 7**). After 13 days, nitrogen limitation alone (+S/−N medium) reduces short-chain aliphatic glucosinolate levels to 8%, long-chain aliphatic glucosinolate levels to 20% and indolic glucosinolate levels to 15% compared to the nutrient-sufficient control group (+S/+N medium). Within the long-chain aliphatic glucosinolates, this reduction was more pronounced for methylthioalkyl glucosinolates (7MTH, 8MTO) than for methylsulfinylalkyl glucosinolates (7MSH, 8MSO) (**Supplementary Table 7**).

Sulfur limitation resulted in an overall reduced accumulation of glucosinolates, but when looking at the three main classes, only the reduced levels of aliphatic glucosinolates were statistically significant. Long-chain aliphatic glucosinolate levels were reduced to 14% and short-chain aliphatic glucosinolate levels to 40% of the nutrient-sufficient control group with similar effects on the individual structures within these classes. Although the levels of total indolic glucosinolates and the levels of NMOI3M were not affected by sulfur limitation, the levels of I3M and 4MOI3M were significantly reduced (**Supplementary Tables 7** and **8**). Almost exclusively for long-chain aliphatic glucosinolates, we observed synergistic effects of sulfur and nitrogen limitation (**Supplementary Table 8**).

Levels of embryo-synthesized glucosinolates decreased over time after transfer, but their turnover rate was not affected by nitrogen or sulfur limitation or the combination thereof (**Figure 4B** and **Supplementary Table 7**). Accumulation of the general turnover metabolite raphanusamic acid was highest under nitrogen sufficient conditions and thus did not follow the turnover of embryo-synthesized glucosinolates (**Figure 4B**). Instead, we found the levels of raphanusamic acid to be positively correlated with total glucosinolate accumulation (**Figure 4C** and **Supplementary Figure 5**). Availability of nitrogen and sulfur significantly affected seedling growth (**Supplementary Figure 6**). Nitrogen limitation showed the strongest negative effect on seedling growth (P < 0.001), resulting in stagnation of growth after eight days. The seedling weight was reduced to 13% compared to the nutrient-sufficient group after 13 days under limiting conditions irrespective of the availability of sulfur, which had a smaller negative effect on seedling weight (P = 0.047; **Supplementary Table 9**). Thus, total endogenous glucosinolate accumulation followed the pattern of seedling growth under nutrient limiting conditions.

### Allyl Glucosinolate Accumulation Is Time- and Dose-Dependent and Promotes the Accumulation of Raphanusamic Acid

**Figure 5:**
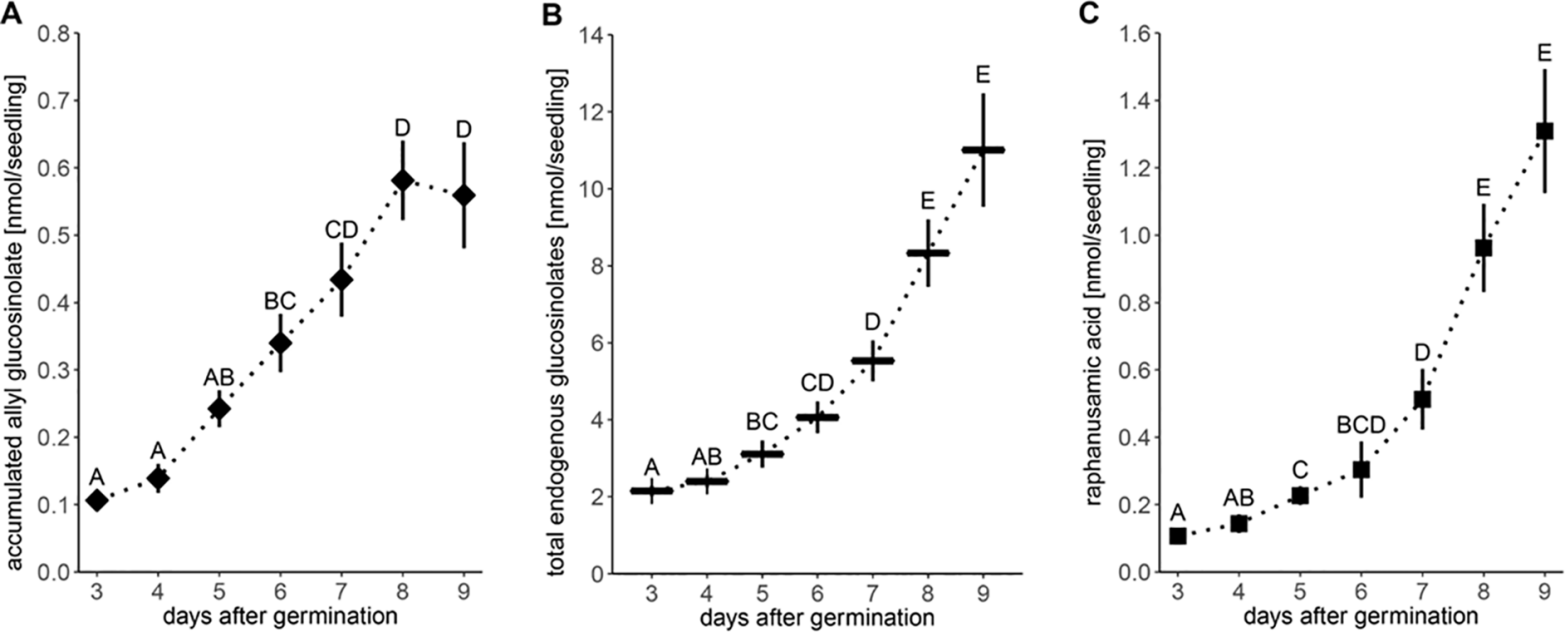
Accumulation of allyl glucosinolate **(A)**, total endogenous glucosinolates **(B)** and raphanusamic acid, **(C)** in *A. thaliana* Col-0 seedlings grown continuously on media containing 50 µM allyl glucosinolate. Depicted are means ± SE (N = 17–19, one experimental round) and letters denote significant difference at the 0.05 level. Details on the statistical analysis including means and standard deviation are provided in **Supplementary Table 10** for allyl glucosinolate, glucosinolate groups and raphanusamic acid.

*A. thaliana* Col-0 does not biosynthesize allyl glucosinolate at any developmental stage (Kliebenstein et al., 2001; Li and Quiros, 2003; Wentzell et al., 2007; Burow et al., 2015). Accordingly, we did not detect allyl glucosinolate in this study unless it had been applied exogenously. Feeding this glucosinolate to Col-0 seedlings therefore makes it possible to experimentally uncouple biosynthesis and turnover (Francisco et al., 2016a). Seedlings grown on medium containing 50 µM allyl glucosinolate steadily accumulated increasing amounts of allyl glucosinolate (**Figure 5A**) and reached levels corresponding to approx. 5% of the total endogenous glucosinolates (**Figure 5B** and **Supplementary Tables 10** and **11**). Notably, raphanusamic acid levels were considerably higher in allyl glucosinolate-treated seedlings. Nine days after germination, seedlings that had grown on allyl glucosinolate-containing medium accumulated 1.3 nmol raphanusamic acid, whereas the control seedlings only accumulated less than 0.02 nmol (**Figures 2B** and **5C**). Feeding of allyl glucosinolate moreover coincided with accumulation of 0.56 nmol allyl glucosinolate per seedling and increased levels of total endogenous glucosinolates (11 nmol/seedling compared to 8 nmol/seedling grown on control medium) nine days after germination, indicating that the turnover of endogenous glucosinolates might contribute to the accumulation of raphanusamic acid at this developmental stage.

**Figure 6:**
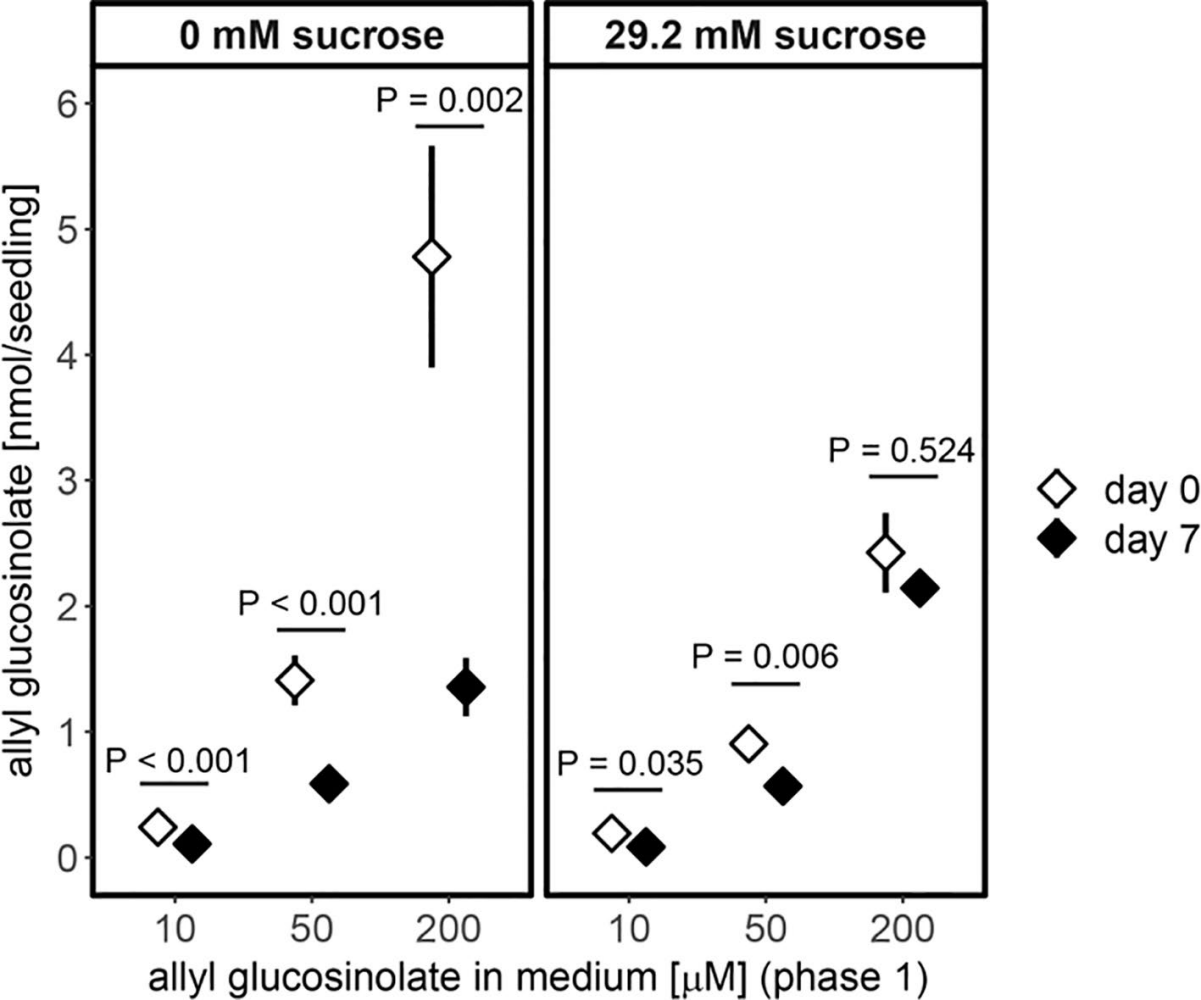
Accumulation of allyl glucosinolates in six-day-old seedlings (white diamond, transfer day) and its turnover after seven days on allyl glucosinolate-free medium (black diamond, end of phase 2) is dependent on the concentration of allyl glucosinolate present in the growth medium (phase 1) and the absence (left panel) and presence of 29.2 mM sucrose (right panel). Depicted are means ± SE, N = 15–18 for 0 mM sucrose (data pooled from two independent experiments) and N = 5–8 for 29.2 mM sucrose treatment (one experiment). Pairwise comparison of day 0 to day 7 within an allyl glucosinolate and sucrose treatment group was performed using Wilcoxon rank sum test (P-value adjustment method: Benjamini & Hochberg). Details on the statistical analysis are provide in **Supplementary Table 11**. For details on the experimental set-up refer to **Supplementary Figure 3**. For the effect of varying nitrogen and sulfur content on the growth medium on allyl glucosinolate turnover refer to **Supplementary Figure 8**. Fresh weight of seedlings grown on nitrogen and/or sulfur sufficient and limiting conditions (phase 2) depending on the allyl glucosinolate concentration in phase 1 on sucrose-free medium is plotted in **Supplementary Figure 9**.

Furthermore, allyl glucosinolate accumulated to higher levels with increasing concentrations of allyl glucosinolate in sucrose-free growth medium (**Figure 6**). The presence of 29.2 mM sucrose in the growth medium decreased the accumulation in seedlings grown on high relative to low allyl glucosinolate concentrations six days after germination (white diamonds, **Figure 6**) and allyl glucosinolate accumulation remained sucrose-dependent over time (**Supplementary Figure 7** and **Supplementary Table 12**). To investigate the turnover of allyl glucosinolate, allyl glucosinolate-containing seedlings were transferred to glucosinolate-free medium with the same sucrose concentration and grown for another seven days (**Figure 6**). Allyl glucosinolate levels were reduced by 54%, 58% and 72% for seedlings treated with 10 µM, 50 µM and 200 µM allyl glucosinolate, respectively, after growing on 0 mM sucrose compared to the levels on the day of transfer (black diamonds, **Figure 6**). In comparison, allyl glucosinolate levels were reduced only by up to 20% for seedlings grown on 29.2 mM sucrose (**Figure 6**) suggesting a reduced rate of allyl glucosinolate turnover. We further investigated the effect of nitrogen and sulfur limitation on allyl glucosinolate turnover, which had only a minor effect within our time frame of investigation depending on the initial allyl glucosinolate concentration applied in the medium (**Supplementary Figure 8** and **Supplementary Table 12**). Seedling weight, however, was significantly affected by both, the concentration of allyl glucosinolate supplied in the accumulation phase (Phase 1, P < 0.001) and nitrogen limitation in the turnover phase (Phase 2, P < 0.001) for 0 mM sucrose treatment (**Supplementary Figure 9** and **Supplementary Table 13**).

**Figure 7:**
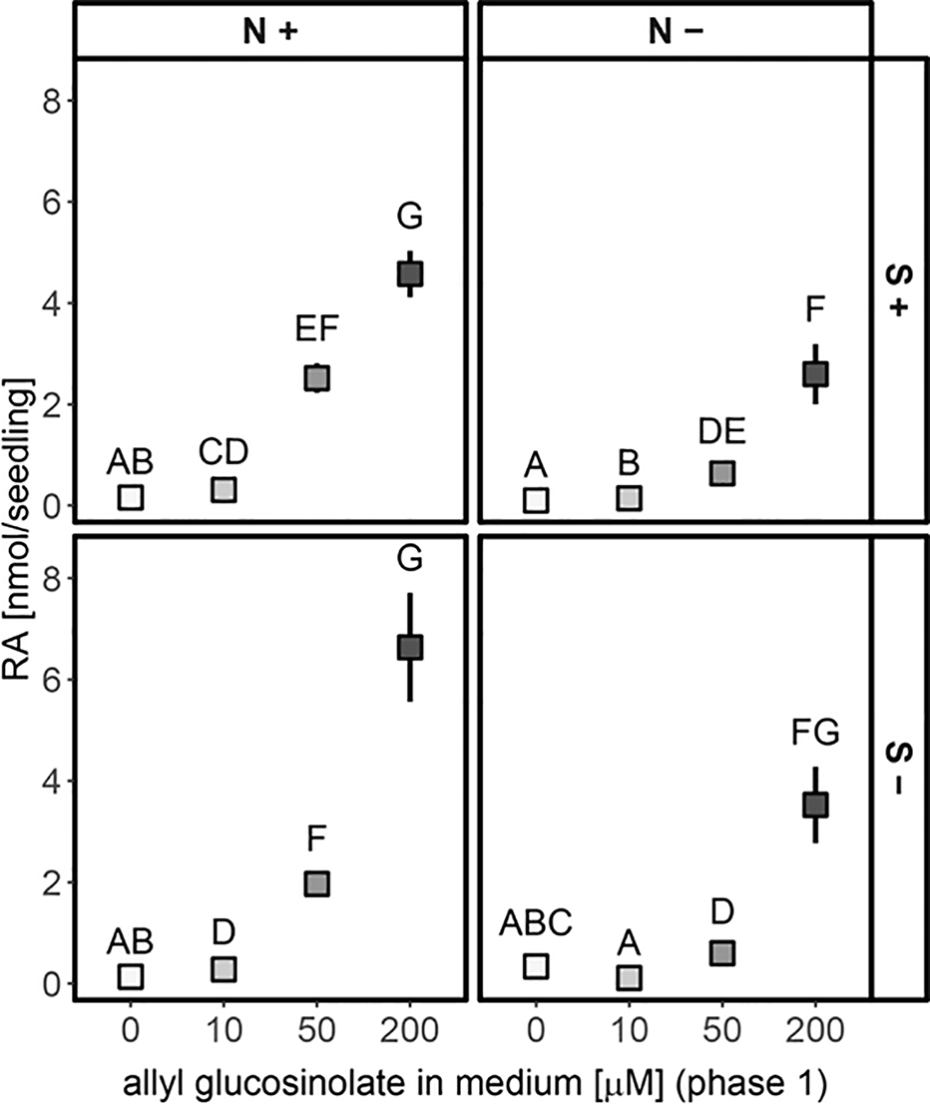
Accumulation of raphanusamic acid in 13-day-old seedlings dependent on the concentration of allyl glucosinolate supplied in the growth medium in the first six days of seedling development (phase 1, grey scale) and the sulfur (S) and nitrogen (N) availability for additional seven days (phase 2) on 0 mM sucrose medium. Depicted are means ± SE, N = 15–18, data pooled from two independent experiments. Letters denote significant differences at the 0.05 level with pairwise comparison using Wilcoxon rank sum test (P-value adjustment method: Benjamini & Hochberg). Details on the statistical analysis are provided in **Supplementary Table 14**. For details on the experimental set-up refer to **Supplementary Figure 3**.

The accumulation of raphanusamic acid was positively correlated with increased allyl glucosinolate accumulation (P(concentration) < 0.001) and nitrogen availability (P(N) < 0.001; **Figure 7**). This effect was stronger for seedlings that had initially grown on high allyl glucosinolate concentrations (P(N * concentration) < 0.001). Although allyl glucosinolate levels were similar across all four media seven days after transfer depending on the initial allyl glucosinolate concentration in Phase 1 (**Supplementary Figure 8**), raphanusamic acid levels varied among media (**Figure 7**). Raphanusamic acid accumulated to amounts expected based on turnover of allyl glucosinolate under nitrogen limiting conditions (−N) if 1 nmol allyl glucosinolate is detected as 1 nmol raphanusamic acid. For nitrogen sufficient conditions (+N), appr. 2-fold higher amounts were detected as could be expected from allyl glucosinolate turnover alone (comparing the difference of days 0 and 7, **Figure 6**, to the levels in **Figure 7**), suggesting that raphanusamic acid also stems from turnover of endogenous glucosinolates in intact plant tissue.

### Allyl Glucosinolate Treatment Alters the Turnover of Embryo-Synthesized Glucosinolates

**Figure 8:**
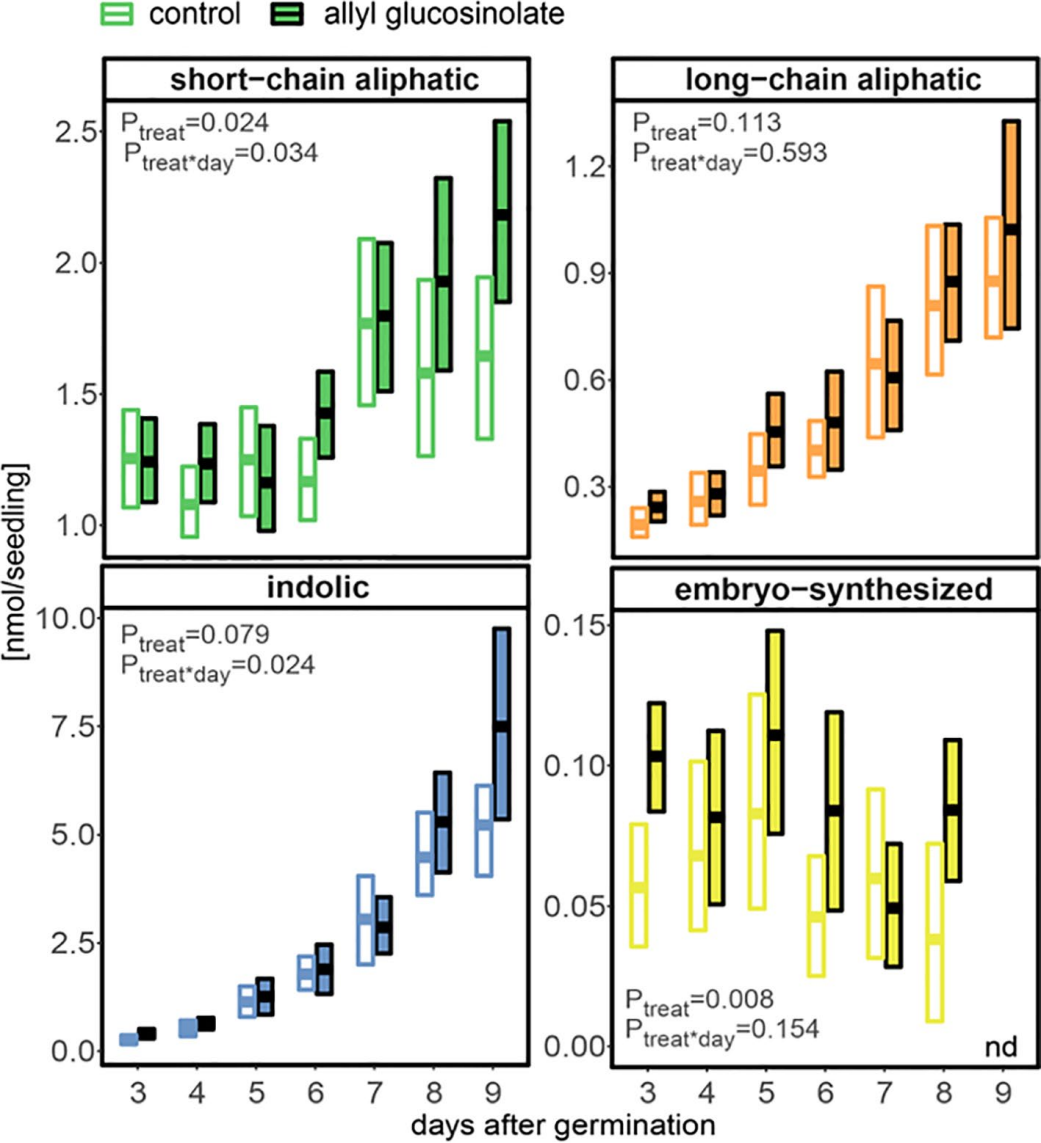
The influence of exogenously applied allyl glucosinolate on endogenous glucosinolate profiles in *A. thaliana* Col-0 seedlings. Seedlings were grown on control (glucosinolate-free) medium (white fill) and medium containing 50 µM allyl glucosinolate (colored fill) for nine days after germination. Plotted are means (bold bar) and 95% confidence interval (box), N = 6–19 (one experimental round). Embryo-synthesized glucosinolates were not detected (nd) in nine-day-old seedlings. Statistical comparison of the control and allyl glucosinolate treatment was performed with a linear model of the following function: aov(analyte ∼ treat*day), with treat = treatment. Details on the statistical analysis including means, standard error and post-hoc tests are in **Supplementary Table 1** for the control treatment and **Supplementary Table 15** for allyl glucosinolate treatment.

In seedlings grown on 50 µM allyl glucosinolate-containing medium, short-chain aliphatic glucosinolates showed the same pronounced increase seven days after germination (**Figures 2A** and **8**), but accumulated to higher levels compared to those in seedlings grown on allyl glucosinolate-free medium (P = 0.024, **Supplementary Table 10**). This was driven by a 30% higher accumulation of 4mtb glucosinolate compared to the control group (**Supplementary Table 15**). Indolic glucosinolates, showing the strongest increase rate at this developmental stage, increased continuously over time and reached 43% higher levels in allyl glucosinolate-treated seedlings than in control seedlings nine days after germination. In contrast, neither total long-chain aliphatic glucosinolates nor any individual glucosinolate in the group was affected by allyl glucosinolate feeding **Figures 2A**, **8**, and **Supplementary Table 15**). Until eight days after germination, allyl glucosinolate-treated seedlings also maintained significantly higher levels of embryo-synthesized glucosinolates compared to the control group (P = 0.008) (**Figure 8**). In this experimental setup, the levels of embryo-synthesized glucosinolates decreased over time until they were not detectable nine days after germination irrespective of the presence of allyl glucosinolate in the medium.

**Figure 9:**
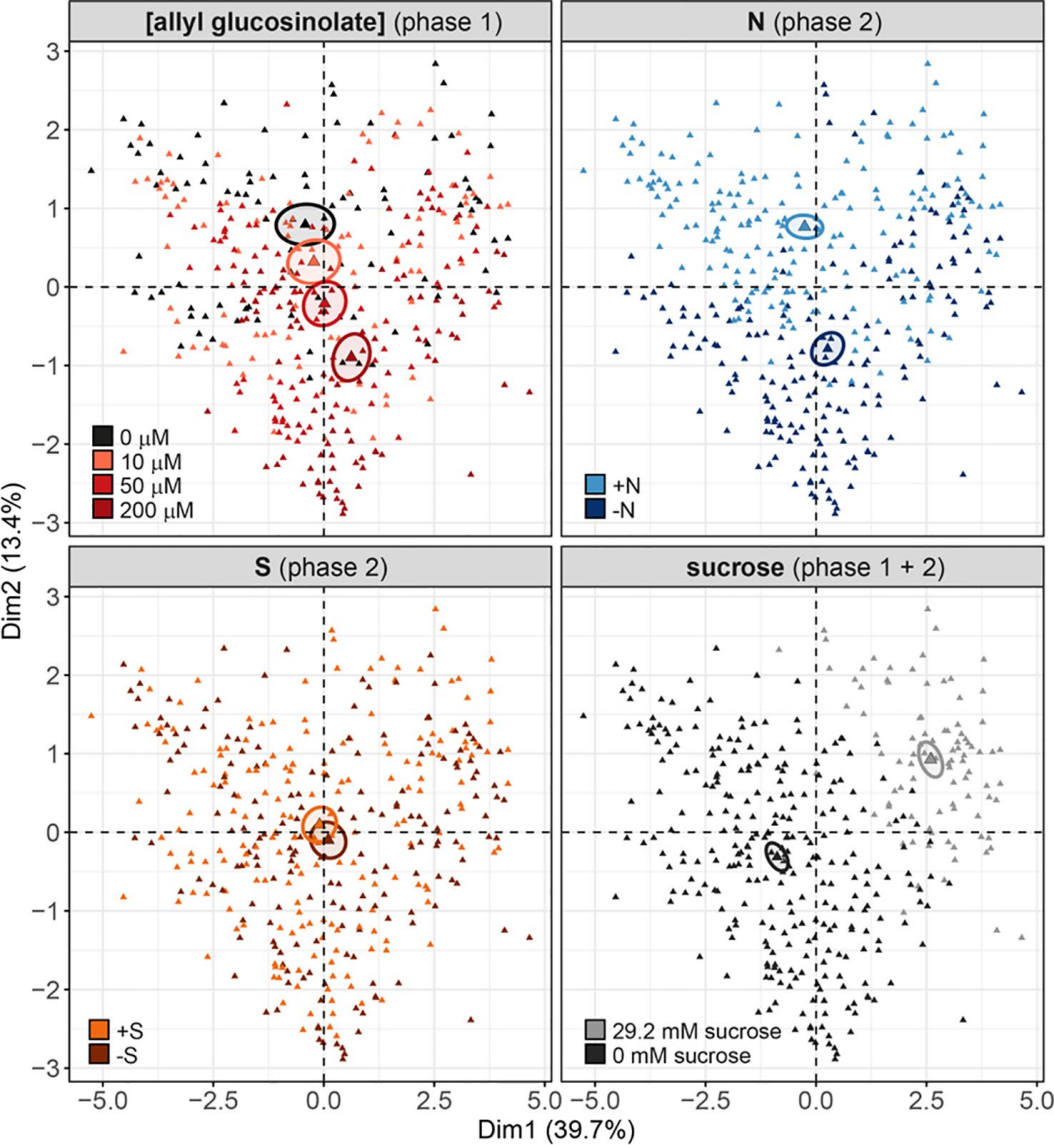
Multiple factor analysis (MFA) of the effects of exogenously feeding allyl glucosinolate (in 0, 10, 50 and 200 μM), varying nitrogen in the growth medium (0.3 mM (−N) and 3 mM (+N)), varying sulfur in the growth medium (0.015 mM (−S) and 1.68 mM (+S) and the effects of the presence (29.2 mM) and absence and sucrose on endogenous glucosinolate levels. Seedlings were grown on medium containing different concentrations of allyl glucosinolate for six days (phase 1) and transferred to glucosinolate-free medium varying in their nitrogen and sulfur content for seven days (phase 2, for details on the experimental set-up refer to **Supplementary Figure 3**). Sucrose treatment was constant over phase 1 and phase 2. Each triangle represents the sum of all individual glucosinolate concentrations of one single seedling, data pooled from two independent experiments. For a list of the glucosinolate concentrations and the statistical analysis by treatment group refer to **Supplementary Tables 16** and **17**. For a list of the contribution of the treatment and glucosinolate classes, and of the contributions of the individual glucosinolates to the separation of dimensions 1 and 2, refer to **Supplementary Figure 10**.

Nine days after germination, seedlings grown on 50 µM allyl glucosinolate-containing medium had accumulated >35% higher total levels of glucosinolates than seedlings grown on control medium (**Figures 2A** and **8**). To compare the effects of allyl glucosinolate feeding to those of nitrogen and sulfur availability, and sucrose on endogenous glucosinolate profiles, we performed a multiple factor analysis (MFA) on a principle component analysis (PCA) on the entirety of the individual glucosinolates detected with our method (for all values and treatment combinations, **Supplementary Table 17**). The analysis was performed on single seedlings that were first grown on various concentrations of allyl glucosinolate with or without the addition of 29.2 mM sucrose (phase 1). Six days after germination, the seedlings were grown for another seven days (in sum, 13-day-old seedlings) on glucosinolate-free media that were sufficient or limiting in nitrogen and/or sulfur and either contained 29.2mM sucrose, or not (phase 2, **Supplementary Figure 3**). These four variations in medium composition (allyl glucosinolate concentration in Phase 1; nitrogen content, sulfur content and sucrose content in Phase 2) were considered as categorical variables in the principal component analysis, and the individual glucosinolates were further divided into groups (as in **Supplementary Table 17**) as continuous variables for the MFA-PCA analysis. Of the treatments, the absence or presence of sucrose (**Figure 9**, bottom right panel) is the dominant factor describing the separation of the data in the first dimension.

Overall, the presence of sucrose in the growth medium correlates with lowered total glucosinolate levels (**Supplementary Tables 16** and **17**) independent of other variable factors in this experiment. The glucosinolates most strongly contributing to this dimension (thus correlating with the effect of sucrose treatment) are 2PE and NMOI3M, followed by C8 aliphatic glucosinolates, embryo-synthesized glucosinolates and short-chain aliphatic glucosinolate to a lesser extent (**Supplementary Figure 10**, bottom left panel). Dimension 2 best describes the effect of nitrogen variation, followed by allyl glucosinolate feeding (**Figure 9**, top panels; **Supplementary Figure 10**, top right panel). In the 0 mM sucrose treatment group, we find that total glucosinolate levels are negatively correlated with allyl glucosinolate concentration affecting aliphatic and indolic glucosinolates (**Supplementary Table 16**). Nitrogen limitation negatively impacts short-chain aliphatic glucosinolate levels dependent on the allyl glucosinolate concentration fed, particularly negatively affecting 4mtb levels (but not raising 4msb levels) (**Supplementary Figure 10**, bottom right panel; **Supplementary Table 17**). Variation in sulfur availability has a negligible effect on the overall changes in in glucosinolate profiles, and this effect could not be resolved in any other dimension described in this model (dimensions 3 and upwards not shown).

## Discussion

### Glucosinolate Biosynthesis and Turnover Are Coordinated According to Nutrient Availability

Steady-state levels of metabolites are determined by the rates of biosynthesis and turnover. Whereas the regulation of the biosynthetic pathways and their regulators has been intensely studied, especially at the transcriptional level, glucosinolate turnover pathways remained largely elusive and their impact on steady-state levels of glucosinolates awaits to be determined. To this end, we investigated the nutrient-driven dynamics of glucosinolate levels during seedling development in *A. thaliana*, i.e. at a developmental stage at which biosynthesis and turnover of glucosinolates coincide. Between three and nine days after germination, the average rate of total glucosinolate accumulation was ca. 1 nmol/day (**Figure 2A**), indicating that the rate of biosynthesis exceeds the rate of turnover at this stage. Embryo-synthesized glucosinolates were detectable at least nine days after germination under our experimental conditions (**Figure 2B**) and generally found to decrease during seedling development (**Figures 2B**, **4B**, and **Supplementary Table 7**) demonstrating turnover of these compounds. Similarly, the levels of allyl glucosinolate exogenously applied during the first days of seedling development decreased after the seedlings had been moved to allyl glucosinolate-free medium (**Figure 6**). Although, we cannot determine the relative impact of biosynthesis and turnover on those glucosinolate structures that are *de novo* synthesized in the seedlings, our data confirm previous findings (Petersen et al., 2002; Brown et al., 2003) that seedlings possess the enzymatic machinery to metabolize glucosinolates.

Metabolic processes, growth and development in *A. thaliana* seedlings are regulated by nutrient availability, for example a balanced carbon to nitrogen ratio (Martin et al., 2002). The addition of 29.2 mM (1 %) sucrose to the growth medium with sufficient concentrations of sulfur and nitrogen alters glucosinolate profiles (**Figure 3**) (Gigolashvili et al., 2007; Francisco et al., 2016a). Under our experimental conditions, external sucrose had a stronger effect on the dynamics of long-chain aliphatic and indolic glucosinolate accumulation than on short-chain aliphatic glucosinolates, without changes in glucosinolate composition within these three groups (**Figure 3A** and **Supplementary Table 3**). In twelve-day-old seedlings, sucrose and glucose led to higher glucosinolate accumulation in roots and shoots, whereas fructose treatment did not result in significant changes in glucosinolate content (**Figure 3B**). Although the expression of *MYB28*, a positive regulator of aliphatic glucosinolate biosynthesis, is short-term inducible by glucose (Gigolashvili et al., 2007), long term exposure to external carbon sources appears to affect overall glucosinolate content by regulating seedling growth (**Supplementary Figure 4**) without fine-tuning glucosinolate composition.

Sucrose treatment further affected accumulation of the exogenous allyl glucosinolate, which could reflect a negative effect of sucrose on allyl glucosinolate uptake or a positive effect on turnover (**Figure 6**). When we subsequently followed the turnover of allyl glucosinolate after transfer of the seedlings from plates containing 200 µM allyl glucosinolate to allyl glucosinolate-free media, lower amounts were metabolized in seedlings kept on sucrose-containing plates. This observation suggests a lower turnover rate of allyl glucosinolate in the presence of sucrose and shows that sucrose treatment regulates not only glucosinolate biosynthesis but also turnover.

Also sulfur and nitrogen limitation negatively affected the accumulation of all *de novo* synthesized glucosinolate classes (indolic, short- and long-chain aliphatic glucosinolates). Under nitrogen limiting conditions, the strongest fold-reduction was detected for short-chain aliphatic glucosinolates, which are, however, less abundant than indolic and long-chain aliphatic glucosinolates at this developmental stage (**Figure 4A**). In contrast, sulfur limitation had the strongest negative impact on long-chain aliphatic glucosinolates. Although nitrogen has the more pronounced quantitative effect on glucosinolate levels, both nitrogen and sulfur negatively affected *de novo* synthesis of all three groups under conditions of long-term nutrient limitation. In addition to the overall reduction in glucosinolates, sulfur or nitrogen limitation resulted in distinct changes in the levels of individual glucosinolates (**Supplementary Table 7**), indicating that these macronutrients provide a regulatory input different from sucrose.

Because glucosinolates can represent up to 30 % of the total sulfur in a given tissue, they have been discussed as potential source for sulfur under sulfur limiting conditions (Falk et al., 2007). However, in seedlings of *Brassica juncea* and *B. napus* challenged by sulfate deprivation, sulfur is not mobilized from glucosinolates (Aghajanzadeh et al., 2014). Similarly, neither sulfur nor nitrogen limitation had a significant effect on the turnover of embryo-synthesized glucosinolates under our experimental conditions (**Figure 4**) or on the turnover of exogenously fed allyl glucosinolate (**Supplementary Figure 8**). Although sulfur and nitrogen availability do not seem to change glucosinolate turnover rates in Brassicaceae seedlings, this may nevertheless happen at later developmental stages.

### Exogenously Applied Allyl Glucosinolate Is Metabolized and Fine-Tunes Glucosinolate Biosynthesis and Turnover

When grown on medium containing allyl glucosinolate, *A. thaliana* seedlings gradually accumulated allyl glucosinolate until eight days after germination in a dose-dependent manner (**Figures 5A** and **6**). Although exogenously applied glucosinolates have previously been shown to be transported between root and shoot in the same way as endogenous glucosinolates and to undergo further side chain modification by the glucosinolate biosynthetic machinery (Li and Quiros, 2003; Andersen et al., 2013; Burow et al., 2015; Francisco et al., 2016a; Malinovsky et al., 2017), it cannot be ruled out that an unknown proportion of allyl glucosinolate provided in the medium will encounter myrosinase-catalyzed breakdown. Nevertheless, the addition of sucrose to the growth medium quantitatively affects allyl glucosinolate accumulation depending on seedling development (**Figure 6** and **Supplementary Figure 7**) (Francisco et al., 2016a). Exogenously applied allyl glucosinolate further affects the endogenous glucosinolate profile (**Figures 5** and **8**) (Francisco et al., 2016a). Short -chain aliphatic and indolic glucosinolates accumulated to higher levels in seedlings grown on allyl glucosinolate compared to seedlings grown on allyl glucosinolate-free medium, although this trend was not significant for long-chain aliphatic glucosinolates (**Figure 8**). This further supports the positive regulatory effect of allyl glucosinolate on the biosynthesis of glucosinolates from methionine and tryptophan (Francisco et al., 2016a).

The presence of sucrose resulted in decreased accumulation of allyl glucosinolate depending on the initial allyl glucosinolate concentration in the growth medium (**Figure 6**), which is likely due to a lower uptake rate of allyl glucosinolate as discussed above. In the absence of external sucrose, seedlings grown on allyl glucosinolate-containing medium maintained higher levels of the embryo-synthesized glucosinolates (**Figure 8**). Likewise, allyl glucosinolate feeding did not lead to altered levels of the short-chain aliphatic 4-methylsulfinylbutyl glucosinolate predominantly present in adult plants, but resulted in altered levels of 4mtb glucosinolate depending on seedling age, illustrating the fine-tuning effect of allyl glucosinolate on the glucosinolate profile in seedlings. Because a substantial proportion of 4mtb but not 4msb glucosinolate in seedlings is derived from the seed, we cannot conclude whether allyl glucosinolate affects biosynthesis and/or turnover at this developmental stage.

Structure-specific effects on growth and development have also been shown for the structurally unrelated 3ohp glucosinolate, which belongs to the embryo-synthesized glucosinolates in *A. thaliana* Col-0. 3ohp glucosinolate was recently shown to inhibit seedling root growth due to its signaling function *via* genes in the sugar sensing Target Of Rapamycin (TOR) pathway (Malinovsky et al., 2017). Dose-dependent effects on root growth have further been shown for the glucosinolate breakdown products indole-3-carbinol and allyl isothiocyanate, although at much higher concentrations and in case of indole-3-carbinol through auxin signaling (Katz et al., 2015; Urbancsok et al., 2017). Based on these observations, we propose a model in which the glucosinolate composition and the metabolic status coordinately feedback regulate glucosinolate profiles and seedling development.

### Raphanusamic Acid Levels Reflect the Levels of Endogenous Glucosinolates

Turnover of glucosinolates *via* the formation of isothiocyanates and their conjugation with glutathione yields raphanusamic acid (Bednarek et al., 2009; Piślewska-Bednarek et al., 2018). Although glucosinolates are predominantly metabolized to simple nitriles in homogenates of *A. thaliana* seedlings (Wittstock et al., 2016b), we monitored the levels of raphanusamic acid as potential indicator of glucosinolate turnover during seedling development and indeed detected increasing amounts from five days after germination (**Figure 2B**). Over time, the kinetics of raphanusamic acid accumulation did, however, not follow the turnover of the embryo-synthesized glucosinolates (**Figures 2** and **4B**). Raphanusamic acid levels were instead correlated with the accumulation of total glucosinolate levels (**Figure 4C**). Considering that one week after germination, the indolic glucosinolate NMOI3M constitutes >40% of total glucosinolates and accumulates at high rate (**Supplementary Table 1**), raphanusamic acid may at least partially reflect the biosynthetic rate of indolic or possibly of total glucosinolates.

In support of this hypothesis, higher levels of raphanusamic acid repeatedly coincided with high flux through the glucosinolate biosynthetic pathways. The levels of raphanusamic acid and glucosinolates were reduced under nitrogen and sulfur limiting conditions (**Figure 4**). Further, the accumulation of allyl glucosinolate over time positively correlated with raphanusamic acid, an effect that persisted seven days after the seedlings had been transferred to allyl glucosinolate-free media (**Figure 7**). These increased levels of raphanusamic acid upon and after allyl glucosinolate feeding may stem from allyl glucosinolate itself but may also reflect an allyl glucosinolate-mediated increased biosynthetic rate. Because raphanusamic acid can possibly be formed form all glucosinolates irrespective of their side chain chemistry, it cannot carry glucosinolate structure-specific regulatory information. Nevertheless, this intermediate of glucosinolate metabolism could serve as checkpoint allowing the plant to measure the flux through the glucosinolate biosynthetic and turnover pathways and thereby to dynamically adjust glucosinolate levels to internal and external signals.

## Data Availability Statement

All datasets generated for this study are included in the article/**Supplementary Material**.

## Author Contributions

VJ and MB design the experiments. KW and SM conducted the sugar dependency experiments. VJ carried out all other experimental work, the statistical analyses and made the figures. VJ and MB wrote the manuscript.

## Funding

This work was supported by The Danish National Research Foundation, DNRF grant 99 (VJ, KW, MB) and Villumfonden, project no. 13169 (VJ, MB).

## Conflict of Interest

The authors declare that the research was conducted in the absence of any commercial or financial relationships that could be construed as a potential conflict of interest.
