# Supplementary material for: Coordination of Glucosinolate Biosynthesis and Turnover Under Different Nutrient Conditions
Source: Front Plant Sci. 2019 Dec 6;10:1560. doi: 10.3389/fpls.2019.01560 (PMC6909823; doi:10.3389/fpls.2019.01560)
Supplement: Supplementary file 1 [file DataSheet_1.zip › Supplementary Figures.pdf]

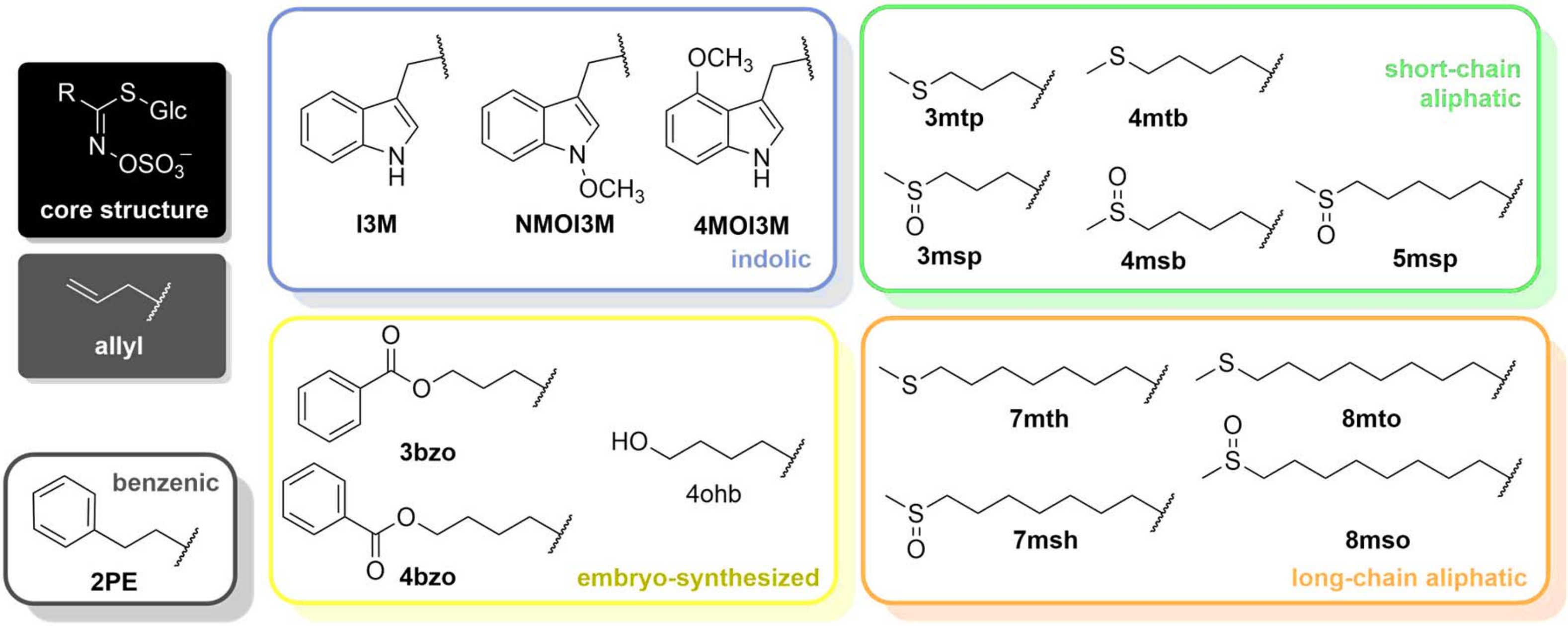

Supplementary Figure 1. The glucosinolate core structure (black box), and the side-chain structures of the exogenously supplied allyl glucosinolate (grey box) and of the glucosinolates detected in *A. thaliana* Col-0 seedlings grouped into indolic (blue), short-chain aliphatic (green), long-chain aliphatic (orange), embryo-synthesized glucosinolates (yellow) and benzenic (grey). Glucosinolate side-chain structures: 2PE, 2-phenylethyl; 3bzo, 3-benzoyloxypropyl; 3msp, 3-methylsulfinylpropyl; 3mtp, 3-methylthiopropyl; 4bzo, 4-benzoyloxybutyl; 4msb, 4-methylsulfinylbutyl; 4mtb, 4-methylthiobutyl; 4ohb, 4-hydroxybutyl; 5msp, 5-methylsulfinylpentyl; 7msh, 7-methylsulfinylheptyl; 7mth; 7-methylthioheptyl; 8mso, 8-methylsulfinyloctyl; 8mto, 8-methylthiooctyl; I3M, indol-3-ylmethyl; NMOI3M, *N*-methoxyindol-3-ylmethyl; 4MOI3M, 4-methoxyindol-3ylmethyl

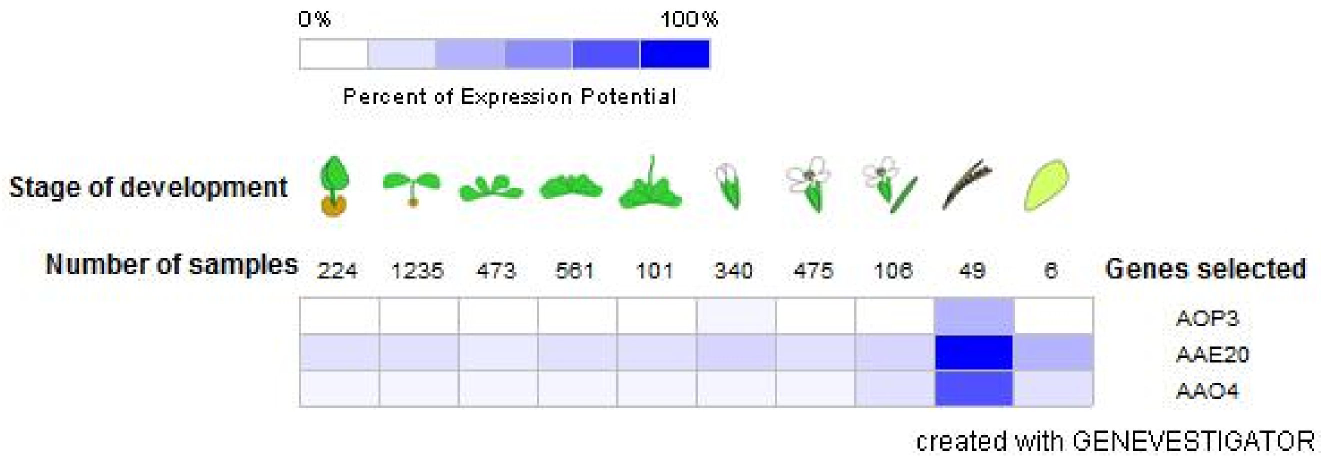

Supplementary Figure 2. Genevestigator analysis of gene expression during *Arabidopsis thaliana* development. The heat map shows expression of genes encoding enzymes involved in the biosynthesis of benzoyloxy glucosinolates: *AOP3* (4g03050), *BZO1* (AAE20, 1g65880), and *AAO4* (1g04580). The 22k array datasets comprises 4048 Col-0 wild type samples collected across all experimental settings. Absolute expression values are depicted in a white-blue scale with colors indicating values normalized to the maximum expression value for each gene. The figure was generated on 01.09.2019.

Reference: Hruz et al. (2008) Genevestigator V3: a reference expression database for the meta-analysis of transcriptomes. *Advances in Bioinformatics*, 420747.

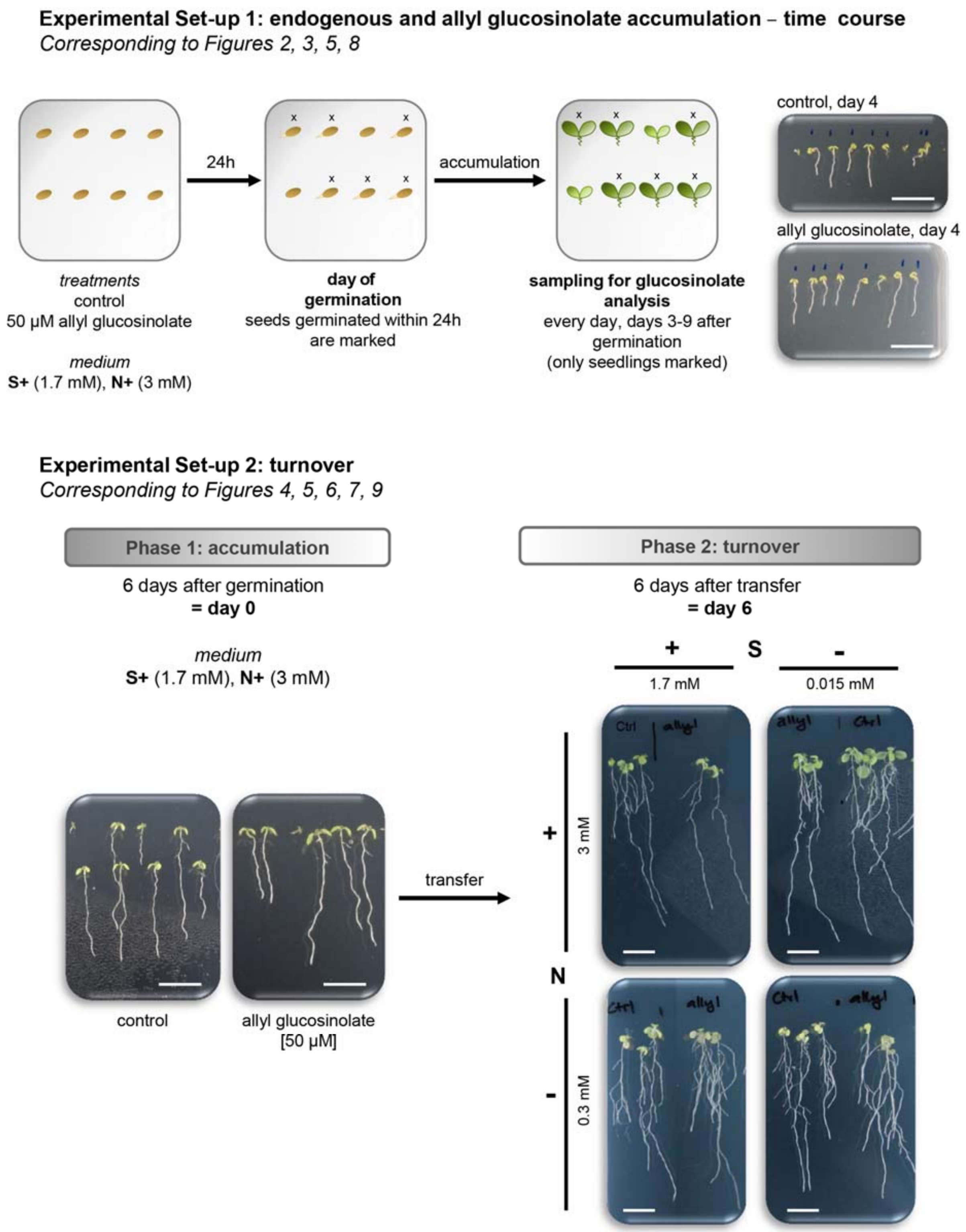

Supplementary Figure 3. The experimental design used for this study. Glucosinolate profiles of seedlings that grew continuously on plates containing 0 µM (control) or 50 µM allyl glucosinolate on sulfur and nitrogen sufficient medium (1.68 mM S, 3 mM N, 0 mM sucrose) were investigated until nine days after germination (**Experimental Set-up 1**). Germination was scored 24 h after plates have been placed in the climate chamber and seedlings not germinated were excluded from the study. A photograph of seedlings four days after germination on control and allyl glucosinolate treatment is depicted on the right, the white bar represents 1cm. In **Experimental Set-up 2** the turnover of exogenously applied allyl glucosinolate was investigated. In **phase 1,** seedlings were grown on plates containing allyl glucosinolate (concentration as described in the figures) and experimental procedures were followed as described in Experimental Set-up 1. Six days after germination, seedlings were transferred to glucosinolate-free medium sufficient (+) or limiting (-) in nitrogen (N) or sulfur (S).

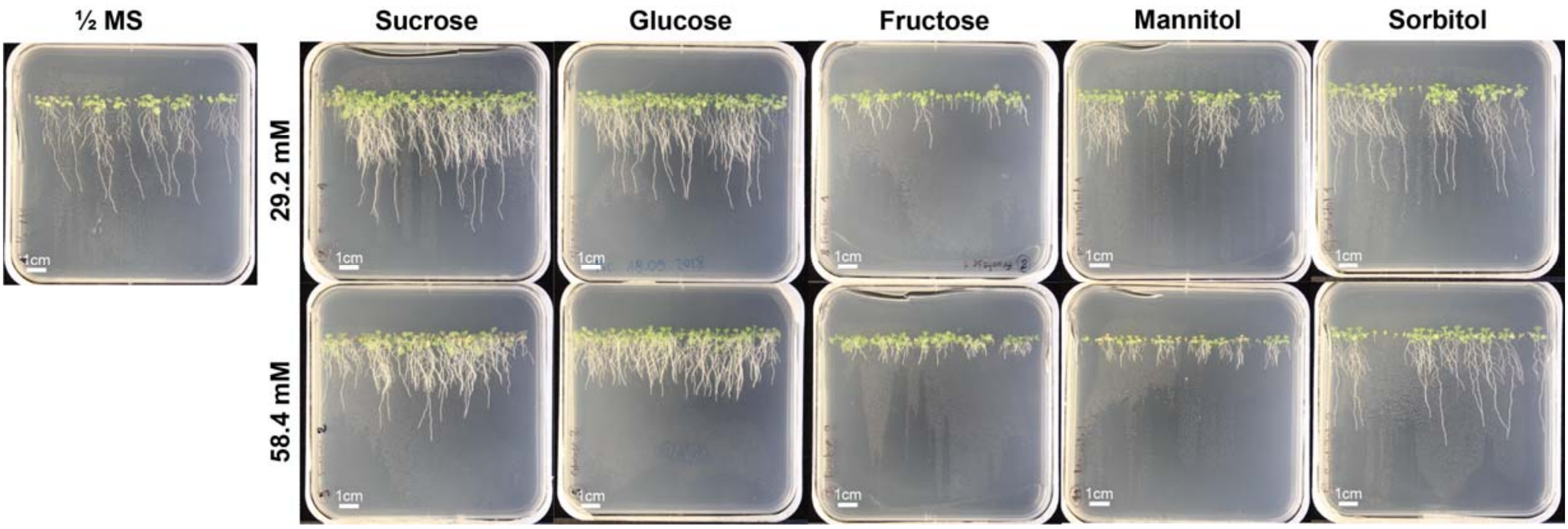

Supplementary Figure 4. Twelve days-old seedlings on ½ MS medium containing different sugars at two concentrations. For glucosinolate contents refer to Figure 3B.

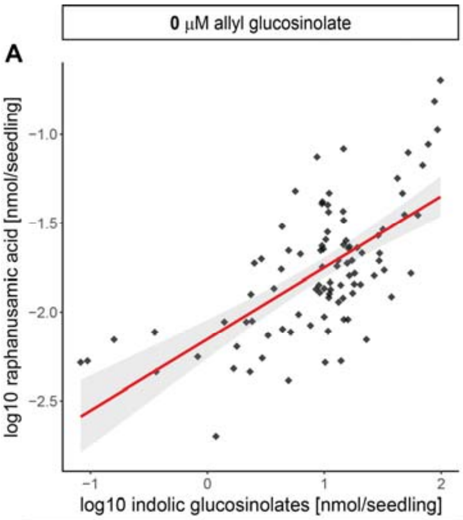

	estimate	SE	t value	P
**intercept**	-2.152	0.055	-39.326	< 0.001
**slope**	0.402	0.048	8.311	< 0.001
residual standard error (SE): 0.281 on 93 df				
**adjusted R-squared: 0.42**				
F-statistic: 69.07 on 1 and 93 df				

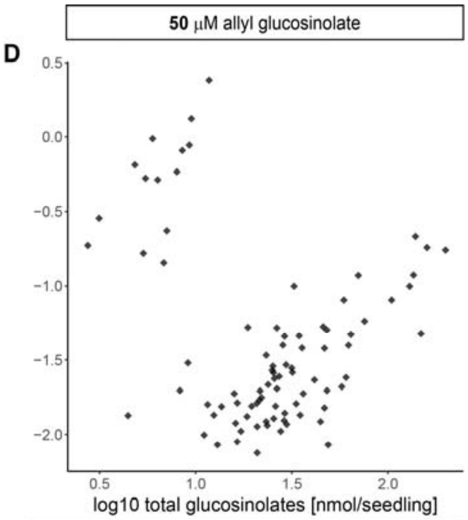

	estimate	SE	t value	P
**intercept**	-0.899	0.23	-3.9	< 0.001
**slope**	-0.353	0.161	-2.197	0.031
residual standard error (SE): 0.581 on 84 df				
**adjusted R-squared: 0.043**				
F-statistic: 4.827 on 1 and 84 df				

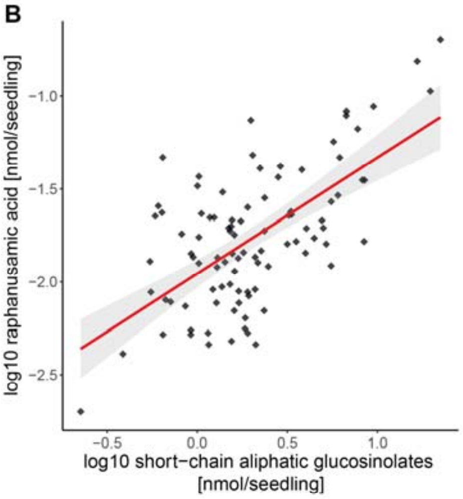

	estimate	SE	t value	P
**intercept**	-1.956	0.038	-51.59	< 0.001
**slope**	0.625	0.079	7.933	< 0.001
residual standard error (SE): 0.286 on 93 df				
**adjusted R-squared: 0.397**				
F-statistic: 62.93 on 1 and 93 df				

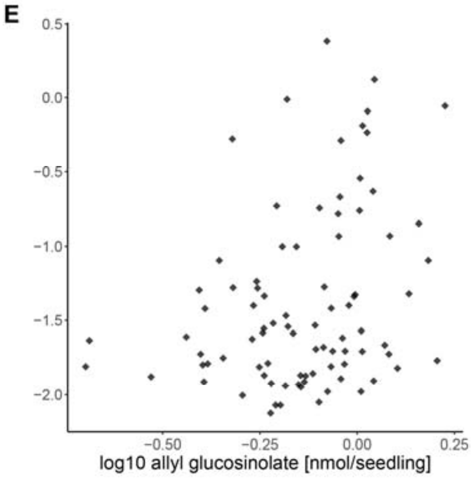

	estimate	SE	t value	P
**intercept**	-1.245	0.077	-16.04	< 0.001
**slope**	1.005	0.342	2.94	0.004
residual standard error (SE): 0.569 on 84 df				
**adjusted R-squared: 0.083**				
F-statistic: 8.645 on 1 and 84 df				

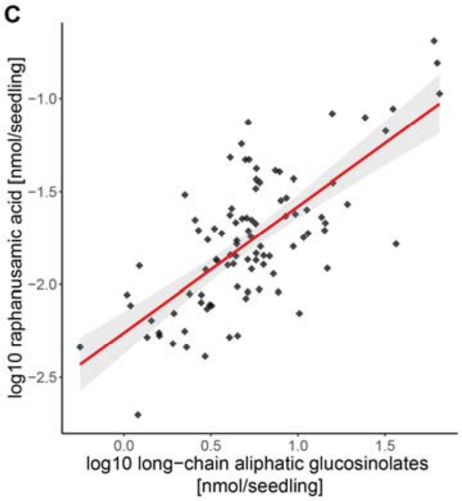

	estimate	SE	t value	P
**intercept**	-2.26	0.057	-39.597	< 0.001
**slope**	0.679	0.069	9.803	< 0.001
residual standard error (SE): 0.260 on 93 df				
**adjusted R-squared: 0.503**				
F-statistic: 96.1 on 1 and 93 df				

Supplementary Figure 5. Correlations of raphanusamic acid to levels of indolic **(A)**, short-chain aliphatic **(B)** and long-chain aliphatic glucosinolates **(C)** for seedlings grown on glucosinolate-free medium, and correlations of raphanusamic acid to total glucosinolate levels **(D)** and accumulated allyl glucosinolate **(E)** for seedlings grown on medium supplemented with 50 μM allyl glucosinolate. Results of the linear model lm(logRA~logGLS) with RA, raphanuamic acid and GLS, glucosinolates are listed in the tables. Depicted are individual samples (dots), the linear correlation (red line) and the 95% confidence interval of the linear correlation (grey shade).

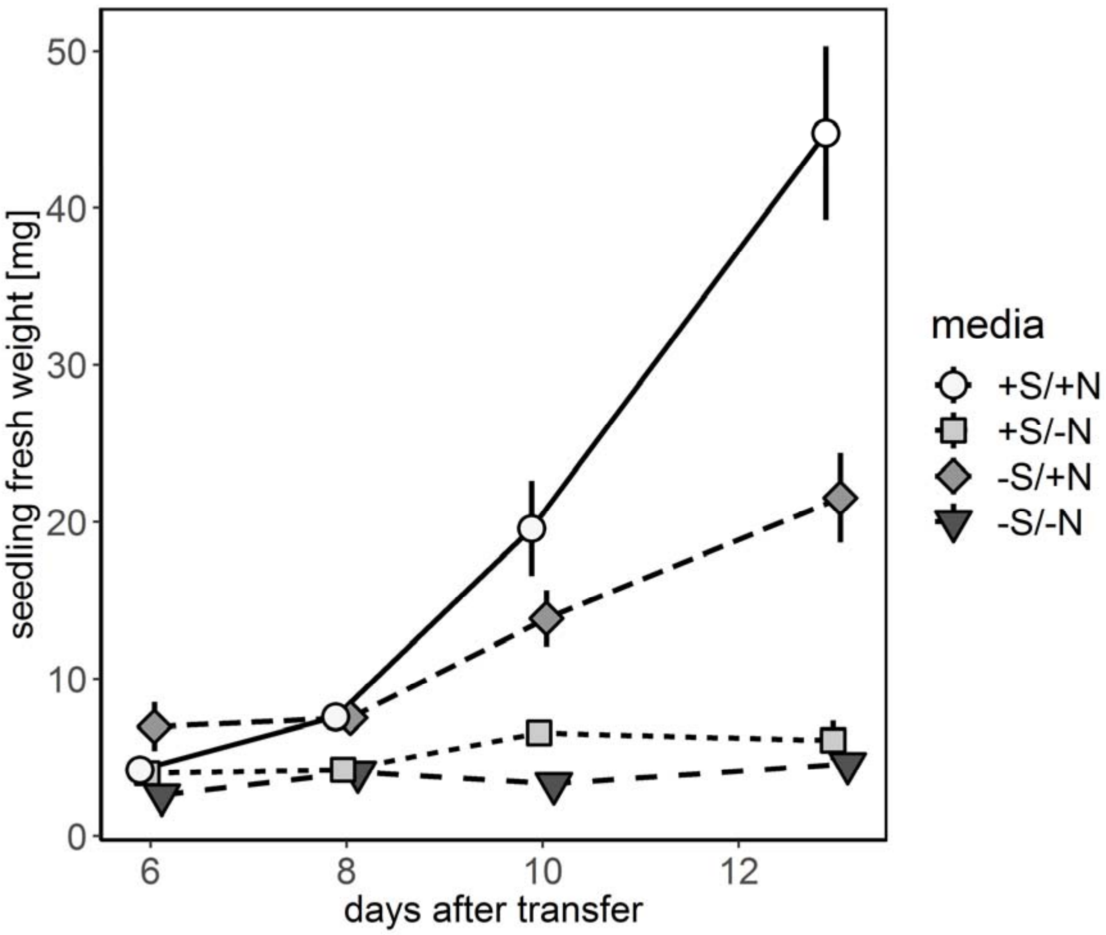

Supplementary Figure 6. Fresh weight of *A. thaliana* Col-0 seedlings grown on medium sufficient or limited in nitrogen (-N) and/or sulfur (-S). Plotted are means ± SE, N = 5-16. Details on the statistical analysis including means and standard deviation are provided in Suppl. Table 9.

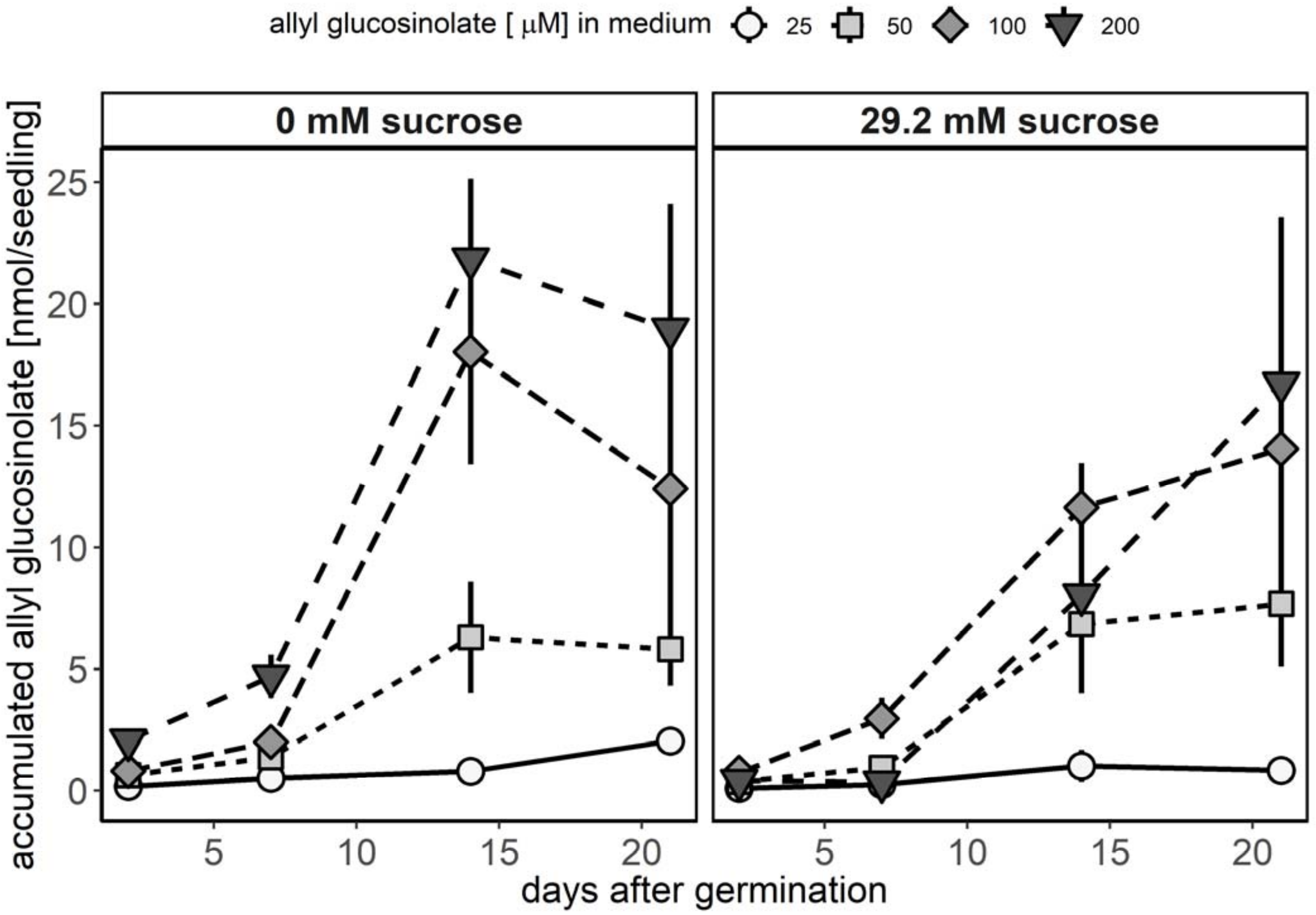

Supplementary Figure 7. Accumulation of allyl glucosinolate in early seedling development depending on the concentration of allyl glucosinolate supplied in the growth medium (grey shade) and presence of sucrose (panels). Depicted are means ± SE (N = 5-8) and details on the statistical analysis are provided in Suppl. Table 12.

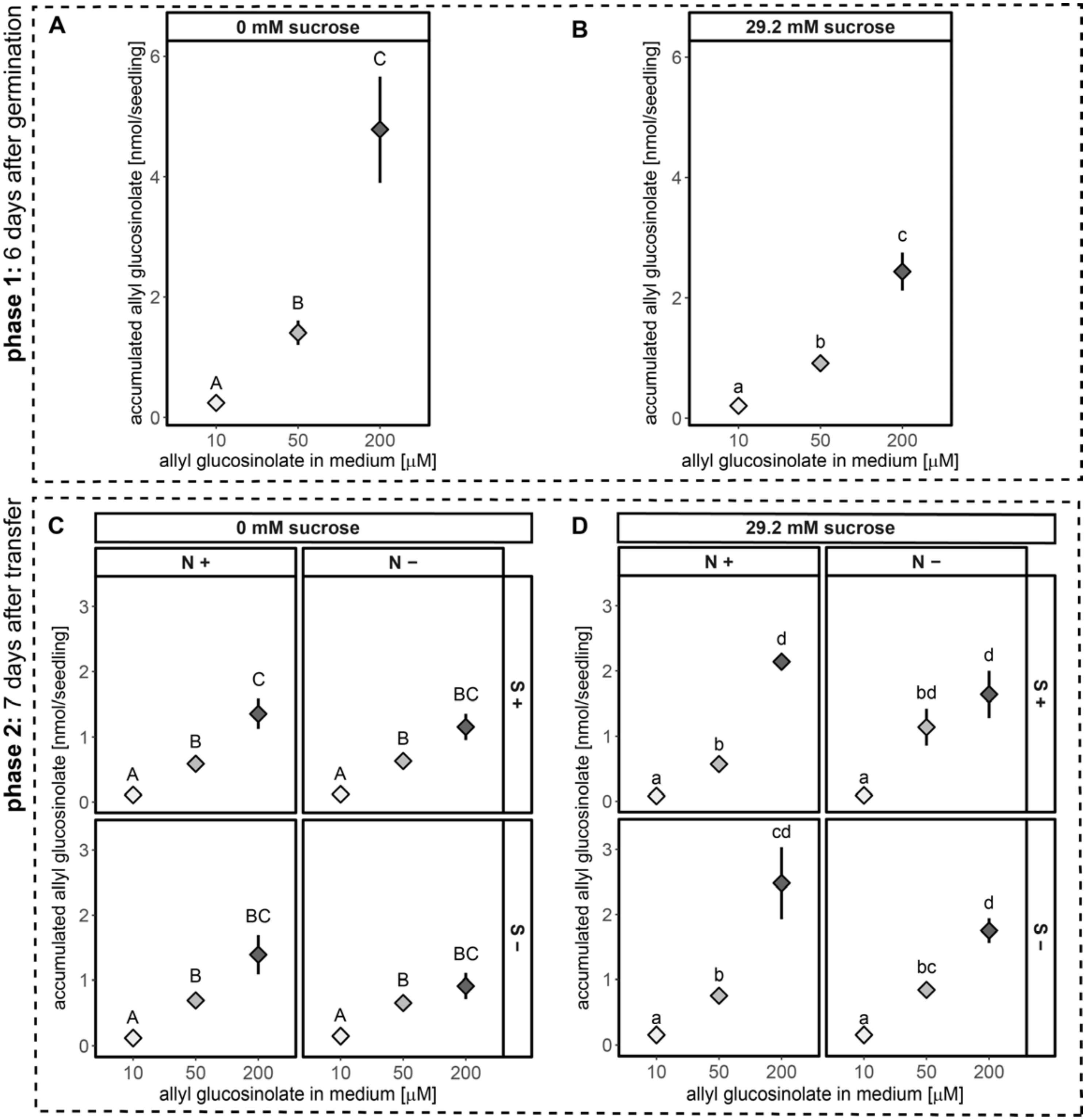

Supplementary Figure 8. Accumulation of allyl glucosinolates in six-day-old seedlings dependent on the concentration of allyl glucosinolate supplied in the growth medium (phase 1, top panel) and the absence **(A)** and presence of 29.2 mM sucrose **(B)**. Levels of allyl glucosinolate after seedlings have been transferred to media with sufficient or limiting sulfur (S) and nitrogen (N) concentrations for seven days (phase 2, bottom panel) dependent on the absence **(C)** and presence of 29.2 mM sucrose **(D)**. Depicted are means ± SE, N = 15-18 for 0 mM sucrose (**A** and **C**, data pooled from two independent experiments) and N = 5-8 for 29.2 mM sucrose treatment (**B** and **D**). Letters denote significant differences within a phase and within sucrose treatment group at the 0.05 level with pairwise comparison using Wilcoxon rank sum test (P-value adjustment method: Benjamini & Hochberg). Details on the statistical analysis are provide in Suppl. Table 11.

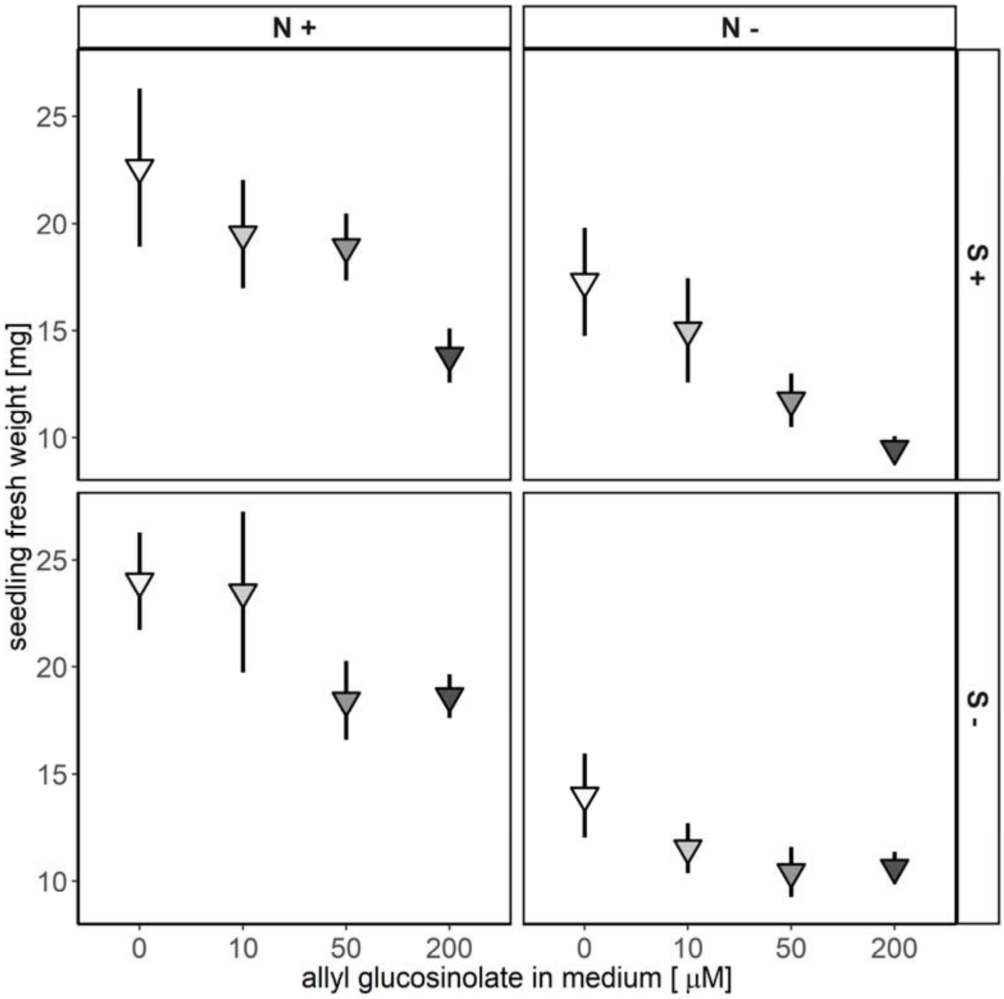

Supplementary Figure 9. Fresh weight of *A. thaliana* Col-0 seedlings grown on different concentration of allyl glucosinolate (grey shade) for six days (phase 1) and on media limited in nitrogen (-N) and/or sulfur (-S) for seven days (phase 2). Plotted are means ± SE (N = 12-14). Details on the statistical analysis including means and standard deviation are provided in Suppl. Table 13.

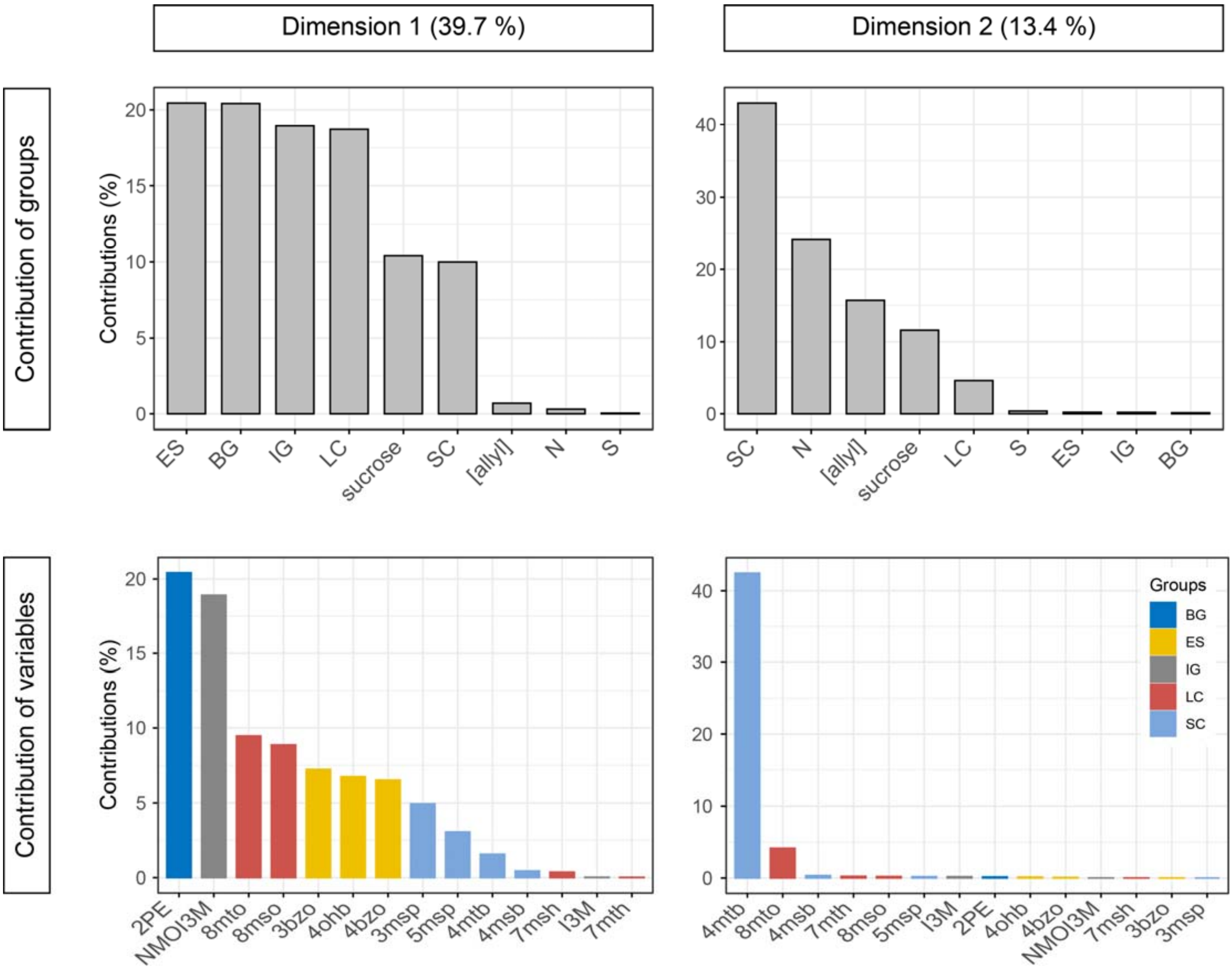

Supplementary Figure 10. The contribution of the treatment and glucosinolate classes (= factor groups, top panels) and the contributions of the individual glucosinolates (= variables, bottom panels) to the separation of dimensions 1 and 2 in the multiple factor analysis (MFA, Figure 9). BG, benzenic glucosinolates; ES, embryo-synthesized glucosinolates; IG, indolic glucosinolates; LC, long-chain aliphatic glucosinolates; N, nitrogen content; S, sulfur content; SC, short-chain aliphatic glucosinolates; [allyl], allyl glucosinolate concentration; for the abbreviations of glucosinolates, refer to Suppl. Figure 1.
